# Strategies to integrate oral health into primary care: a systematic review

**DOI:** 10.1136/bmjopen-2022-070622

**Published:** 2023-07-05

**Authors:** Bradley Christian, Ajesh George, Prabhakar Veginadu, Amy Villarosa, Yuka Makino, Warrick Junsuk Kim, Mohd Masood, Rachel Martin, Yuriko Harada, Maria Carmela Mijares-Majini

**Affiliations:** 1 Population Oral Health, School of Dentistry, The University of Sydney, Sydney, New South Wales, Australia; 2 Australian Centre for Integration of Oral Health, School of Nursing & Midwifery, Western Sydney University, Liverpool, New South Wales, Australia; 3 Ingham Institute for Applied Medical Research, Liverpool, New South Wales, Australia; 4 Menzies School of Health Research, Alice Springs, Northern Territory, Australia; 5 National Centre for Epidemiology and Population Health, The Australian National University, Canberra, Australian Capital Territory, Australia; 6 Noncommunicable Diseases Team, World Health Organization Regional Office for Africa, Brazzaville, Congo; 7 World Health Organization Regional Office for the Western Pacific, Manila, Philippines; 8 Department of Rural Clinical Sciences, La Trobe University - Bendigo Campus, Bendigo, Victoria, Australia; 9 Melbourne Dental School, The University of Melbourne, Melbourne, Victoria, Australia; 10 Oral Health Programme, Noncommunicable Diseases Department, World Health Organization, Geneva, Switzerland

**Keywords:** Health policy, Organisation of health services, PUBLIC HEALTH

## Abstract

**Objectives:**

Integration of oral health into primary care has been proposed as a primary healthcare approach for efficient and sustainable delivery of oral health services, and the effective management of oral diseases. This paper aimed to synthesise evidence on the effectiveness of strategies to integrate oral health into primary care.

**Design:**

Systematic review.

**Data sources:**

MEDLINE, CINAHL, Embase, Scopus, ProQuest, Cochrane and Google Scholar were searched without date limits until the third week of June 2022. Reference lists of eligible studies were also searched. Experts in the field and existing professional networks were consulted.

**Eligibility criteria:**

Only studies that evaluated integration strategies were included in the review. Eligibility was restricted to English language studies published in academic peer-reviewed journals.

**Data extraction and synthesis:**

Two reviewers independently extracted data and performed the risk of bias assessments. A narrative synthesis approach was used to report review findings. Heterogeneity among included studies precluded a meta-analysis.

**Results:**

The search identified 8731 unique articles, of which 49 were included in the review. Majority of the studies explored provision of oral healthcare by primary care professionals in primary care settings, where integration was primarily via training/education and/or policy changes. Most studies reported results favouring the integration strategy, such as improvements in referral pathways, documentation processes, operating efficiencies, number of available health staff, number of visits to non-dental primary care professionals for oral health issues, proportion of children receiving fluoride varnish applications/other preventive treatment, proportion of visits to an oral health professional and dental caries estimates.

**Conclusion:**

The findings from this review demonstrate that the majority of identified strategies were associated with improved outcomes and can be used to inform decision-making on strategy selection. However, more research and evaluation are required to identify best practice models of service integration.

**PROSPERO registration number:**

CRD42020203111.

**STRENGTHS AND LIMITATIONS OF THIS STUDY**

- This review was conducted in collaboration with the WHO and in alignment with WHO’s soon-to-be-released Global Oral Health Action Plan to provide programme planners, policy-makers and decision influencers a list of evidence-informed strategies to integrate oral healthcare in the non-dental primary care setting.
- The identified strategies were synthesised according to WHO’s Health System Building Blocks framework to provide a systems perspective for each strategy.
- The study has a particular focus on outcomes and the scientific quality of included studies.
- Generalisability of findings from this review may be limited due to the considerable heterogeneity in the integration strategies, settings, study designs and outcomes assessed among included studies.
- Due to resource constraints, the grey literature was not searched, and as such, some relevant results may have been missed, though the likelihood is very low given that content experts (globally) were consulted for their knowledge of any such activity.

## Introduction

In recent times, oral diseases have been gaining increasing attention due to the immense burden they place on the individual and community. The draft Global Oral Health Action Plan and its monitoring framework is (at the time of writing this review) being finalised after going through a global consultation process. The proposed Global Target 4.1: Oral health in primary care, in the draft action plan, is that 80% of countries will have oral healthcare services provided in primary care settings by 2030.^1^

As reported in Benzian and Beltràn-Aguilar, key challenges to improving oral health are ‘lack of political priority, inadequate technical oral health capacities within governments, a dominance of expensive curative treatment models despite largely preventable diseases, and data limitations for planning and monitoring equitable services. Additional barriers include the dominating private, dentist-centred delivery mode of oral healthcare and half-hearted measures to tackle the commercial determinants of oral health.’ (Benzian and Beltràn-Aguilar, p2)^2^ The 2019 Lancet commissioned series on oral health highlighted key issues in global oral health, including oral healthcare traditionally being siloed from primary care, the failure of the current surgical model of oral healthcare having any impact on the prevalence or incidence of oral diseases and the major global public health burden of oral diseases.^3 4^ The 2017 Global Burden of Disease study showed that in the last 30 years, for both disease prevalence and incidence, dental caries was in the top five diseases contributing to disease burden.^5^

Integration of oral healthcare into primary care has been proposed as a primary healthcare approach, that could potentially reduce healthcare cost, improve access to care, and increase operating efficiencies. Ultimately, it improves patient outcomes and leads to efficient and sustainable delivery of dental services, and effective management of oral diseases.^6^ Several recent WHO advocacy and strategy documents continue to highlight this urgent need for integration of oral health in order to reduce disease burden.^7 8^ In October 2019, the outcome of an informal WHO consultation on oral health, which included representatives from twenty two countries from both the Western-Pacific and South-East Asia regions, was agreement on four priority action areas, of which two were related to the urgent need to integrate essential oral healthcare into primary care.^8^ Advocacy, leadership and partnership is essential to driving the integration agenda and examples of these include the development of the WHO Framework on integrated people centred health services,^9^ the establishment of the National Interprofessional Initiative on Oral Health^10^ in the USA, and the Australian Network for the Integration of Oral Health.^11^

Harnagea *et al*, in their recent scoping reviews,^12 13^ mapped out the literature on strategies for the integration of oral healthcare into primary care, which included theoretical frameworks; descriptions and evaluations of applied models/programmes; policies and strategic plans; and factors that facilitate integration. The strategies identified in these reviews were implemented at various levels of the healthcare system—organisation, professional and patient levels.^12^ A 2019 systematic review by Prasad *et al*,^14^ discussed the evidence base support for primary oral healthcare approaches. While these previous reviews provided a comprehensive overview of the types of integration strategies, the effectiveness of these strategies was not a key focus and as such has not been explored or discussed in detail in these reviews. This constitutes a significant knowledge gap and without which integration strategy selection will be challenging.

Therefore, the purpose of this systematic review was to synthesise the evidence on the effectiveness of strategies that integrate oral health into primary care.

This systematic review presents examples of evidenced based interventions that countries could consider when translating the recent World Health Assembly (WHA) oral health resolution into local policy and action.

### Review objectives

1. To identify strategies to integrate oral health into primary care, that have been implemented and evaluated.
2. To determine whether these strategies were effective.
3. To identify research gaps and provide recommendations for future research on this topic.

## Methods

### Key concepts and definitions

The WHO Operational Framework for Primary Health Care provides definitions for both primary care and primary healthcare.^15^ Primary care being defined as ‘a key process in the health system that supports first-contact, accessible, continued, comprehensive and coordinated patient-focused care;’ and primary healthcare defined as ‘whole-of-society approach to health that aims to maximise the level and distribution of health and well-being through three components: (A) primary care and essential public health functions as the core of integrated health services; (B) multisectoral policy and action; and (C) empowered people and communities.’ (WHO and the UNICEF, p. XIII).^15^ The conceptual basis for this review is situated in primary healthcare as an approach to improving oral health outcomes and reducing the global burden of oral diseases.

An integration strategy may refer to any activity or intervention (or combination of activities) whose purpose is to, directly or indirectly, support the inclusion of oral health in primary care. These activities could include (but are not limited to) policies, guidelines, frameworks, funding mechanisms and insurance schemes, interprofessional training and education, interprofessional practice, common performance indicators and establishing local or international networks to support the integration agenda.

For this review, the effectiveness of a strategy refers to any indicator of success related to the outcome of interest. For example, evidence of uptake and satisfaction for a particular strategy; evidence of interprofessional education having an impact on practice; cost-effectiveness; improved knowledge, attitudes and behaviours; and improved oral health outcomes.

The outcome measures of interest were (but were not limited to): (A) Uptake of guidelines and policies to support the integration of oral health into primary care (B) the fidelity of implementation of guidelines, policy and programmes, (C) provider/clinician related such as scope of practice, improved knowledge, improved efficiencies in service delivery and (D) patient related such as improved oral health outcomes (oral diseases), oral health quality of life, change in oral health related knowledge, behaviours and attitudes, cost savings and increased access to care.

### Search strategy and selection criteria

This systematic review was informed by the framework outlined in the Cochrane Handbook for Systematic Reviews of Interventions^16^ and with the Preferred Reporting Items for Systematic Reviews and Meta-Analyses (PRISMA) guidelines.^17^ To achieve adequate breadth of relevant literature, the following sources were searched and consulted: electronic databases, reference lists, existing networks and experts in the field. MEDLINE was the principal search database for this review. Other databases included CINAHL, Embase, Scopus, ProQuest, Cochrane and Google Scholar. Please see online supplemental file 1 for the list of search terms based on key concepts identified in the review and detailed search strategy (online supplemental tables S1 and S2).

10.1136/bmjopen-2022-070622.supp1 Supplementary data

Decisions on the inclusion and exclusion criteria, to determine study selection, were guided by the review aim and objectives (online supplemental table S3). This was an iterative process that was finalised as familiarity with the literature developed and through periodic meetings of the review team. There were no population-level exclusions for this review. For example, all ages, all ability types and all locations were included. However, only peer-reviewed research studies published in English language were included in the review. Unpublished/grey literature was not included owing to time and resource limitations.

Covidence was used to manage the two-stage article screening process. In stage 1, the title and abstract of each retrieved article were independently screened by two review authors against the inclusion and exclusion criteria. Differences were resolved by consulting a third review author. In stage 2, the full-text screening, each retrieved article was independently screened by two review authors against the inclusion and exclusion criteria, similar to stage 1. Differences were resolved by consulting a third review author.

### Data analysis

Covidence was used for data extraction. Data from the final list of selected studies were extracted into an evidence table designed specifically for this study. Data extraction was performed by one review author (PV) and a proportion verified for accuracy by a second review author (BC). Any discrepancies in data extraction were resolved by discussion. Data extraction captured information such as author(s) details, study design, population and setting, type of integration strategy, outcomes and key results. Studies were organised chronologically.

An assessment of risk bias was conducted for all included studies. The following risk of bias tools were used depending on study design and data collection method: the Cochrane Collaboration Tool for Assessing Risk of Bias in Randomised Trials, the Risk of Bias in Non-Randomised Studies of Interventions and the Critical Appraisal Skills Programme (CASP) qualitative checklist.^18–20^ For cross-sectional studies, we created a checklist using items from the STrengthening the Reporting of OBservational studies in Epidemiology (STROBE) statement,^21^ as available tools were not appropriate to a cross-sectional study design. For both the CASP and STROBE tools, the following self-determined criteria were used for study quality: studies with 7–10 ‘yes’ responses=‘high quality’; those with 5–6 ‘yes’ responses=‘moderate quality’; and those with less than 5 ‘yes’ responses=‘low quality’. The risk of bias for each included study was independently assessed by two review authors and differences were resolved via discussion or, when required, by consulting a third review author. The domains assessed for risk of bias for all study designs were the recruitment of individual participants, deviations from the intended interventions, bias in measurement classification of interventions, missing outcome data, outcome measurement and selection of the reported result. Additionally, for randomised controlled trials (RCTs) the randomisation process and confounders were assessed for risk of bias. Studies were not excluded based on quality.

**Figure 1:**
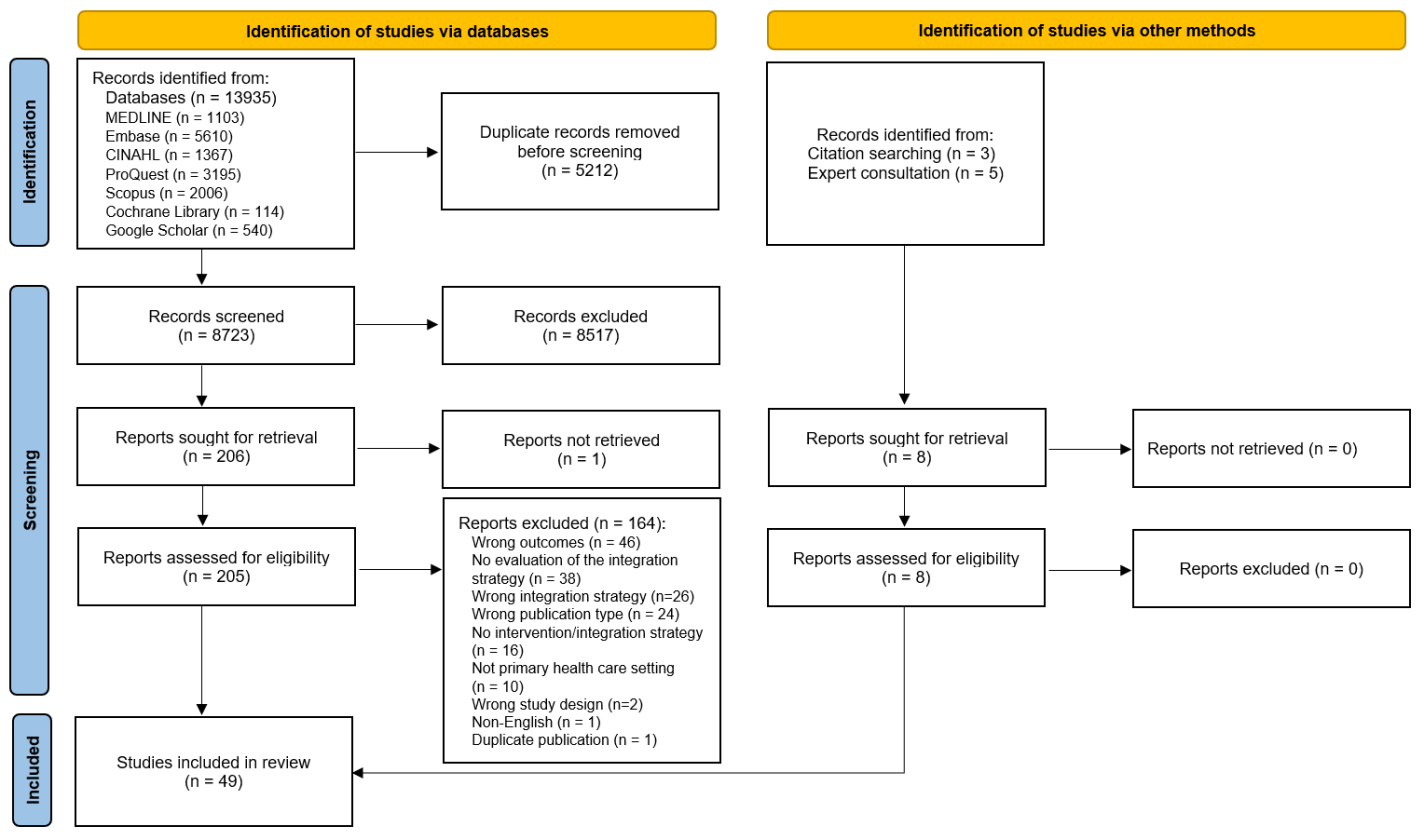
PRISMA flow diagram showing study selection. PRISMA, Preferred Reporting Items for Systematic Reviews and Meta-Analyses.

In line with the WHO Western Pacific Region’s For The Future Vision Paper’s emphasis on orienting and strengthening health systems towards providing integrated people-centred services for NCDs^22^ and the recent WHA oral health resolution,^1^ the data synthesis and structure of presentation of the review results were informed by two key WHO frameworks: (1) WHO’s action framework on universal health coverage (UHC) for the Western Pacific Region and (2) WHO’s action framework on strengthening health systems.^23 24^ This was to ensure alignment of this review with the broader global oral health and regional UHC agenda. The Health System Building Blocks (HSBB) reported in the latter framework provided the scaffolding for reporting and discussing the results for this review. Figure 1 shows the HSBB, the key actions areas of UHC and the relationship between the frameworks. These two frameworks are important in designing coherent and sustainable interventions to improve and sustain the delivery of essential services, especially in primary care settings.^22^

### Patient and public involvement

None.

## Results

**Figure 2:**
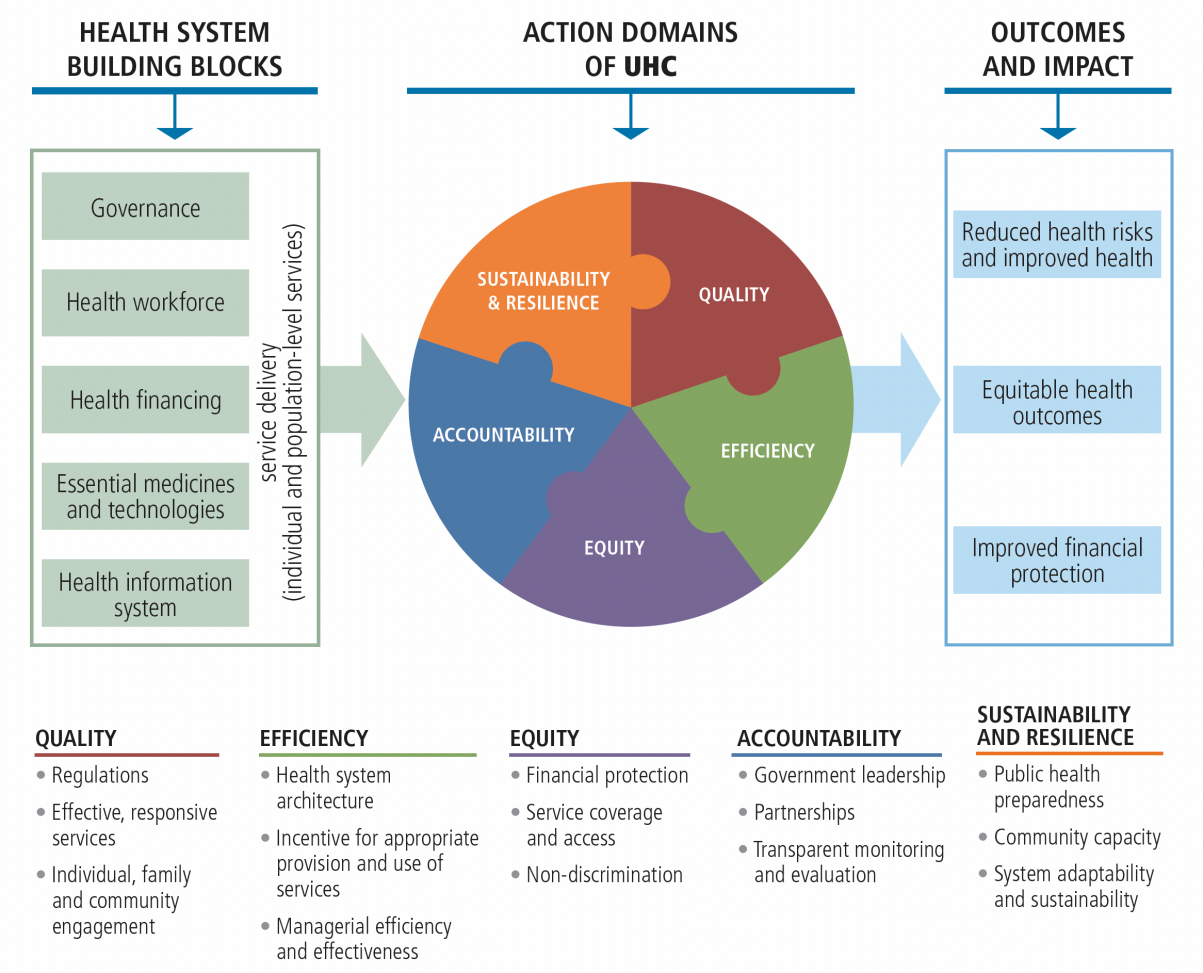
Action areas for the UHC and health systems framework.^23^ UHC, Universal Health Coverage.

The initial search resulted in the identification of 8731 unique articles, of which 213 were assessed for eligibility. On completion of the full text review, 49 studies describing various interventions were included in this review.^25–73^ List of studies excluded during the full text review, along with the reasons, is presented in online supplemental table S4. The PRISMA flow diagram,^17^ shown in figure 2, illustrates the flow of information through the study selection process.

Table 1 presents the general study characteristics, including the study location by WHO region, setting, study design and quality. Majority of the included studies were published in the last decade (n=42) and were predominantly from the USA (n=20), Australia (n=10), Canada (n=5) and Belgium (n=3). By WHO region, over half the studies were conducted in the Americas (n=27). Distribution across the other regions were: Western Pacific (n=11), Europe (n=9), South-East Asia (n=3) and Eastern Mediterranean (n=0). Most studies were conducted in the primary care setting (n=28). Other settings included the community, schools and aged care facilities. To evaluate the strategies, included studies employed qualitative method (n=12), followed by retrospective (n=8), and quasi-experimental/non-randomised trial (n=8). Other designs were pretest/post-test (n=5), mixed-method (n=5), pilot (n=4) and cross-sectional (n=3). Only two included studies were RCTs. The identified studies were assessed to be of high (n=16), moderate (n=19) or low quality (n=14).

**Table 1:** Characteristics of included studies

Author, year, location	WHO region	Study design	Setting*	Quality
Mason, 1994, Scotland^25^	Europe	Pilot	Primary care setting	Low
O'Neil, 2002, Canada^26^	Americas	Cross-sectional	Primary care setting	Low
Lawrence, 2004, Canada^27^	Americas	Cross-sectional	Community	Low
Fallon, 2006, Australia^28^	Western Pacific	Qualitative research	Aged-care facilities	High
Niiranen, 2008, Finland^29^	Europe	Retrospective	Primary care setting	Moderate
Mofidi, 2009, USA^30^	Americas	Retrospective	Community	Moderate
Silk, 2010, USA^31^	Americas	Retrospective	Community	Moderate
Skapetis, 2012, Australia^32^	Western Pacific	Pre–post intervention	Primary care setting	Moderate
Vichayanrat, 2012, Thailand^33^	South-East Asia	Quasi-experimental	Community	Moderate
McKeown, 2014, Canada^34^	Americas	Pre–post intervention	Long-term facilities	Low
Biordi, 2015, USA^35^	Americas	Quasi-experimental	Primary care setting	Low
De Visschere, 2015, Belgium^36^	Europe	Qualitative research	Nursing homes	High
Heilbrunn-Lang, 2015, Australia^37^	Western Pacific	Mixed-method	Primary care setting	Moderate
Kranz, 2015, USA^38^	Americas	Retrospective	Primary care setting	Moderate
McNally, 2015, Canada^39^	Americas	Qualitative research	Long-term facilities	High
Crall, 2016, USA^40^	Americas	Quasi-experimental	Primary care setting	Low
de Mey, 2016, Netherlands^41^	Europe	Pre–post intervention	Primary care setting	Low
Dooley, 2016, USA^42^	Americas	Quasi-experimental	Primary care setting	Low
George, 2016, Australia^43^	Western Pacific	Pre–post intervention	Primary care setting	Moderate
Vece, 2016, USA^44^	Americas	Mixed-method	Primary care setting	Low
Adams, 2017, USA^45^	Americas	Pilot	Primary care setting	High
Duijster, 2017, Cambodia, Indonesia and Lao^46^	Western Pacific/South-East Asia	Non-randomised controlled trial	Schools	Moderate
Kohli, 2017, USA^47^	Americas	Pre–post intervention	Long-term facilities	Low
Mathu-Muju, 2017, Canada^48^	Americas	Qualitative research	Community	High
Sengupta, 2017, USA^49^	Americas	Quasi-experimental	Primary care setting	Moderate
Wright, 2017, Australia^50^	Western Pacific	Retrospective	Aged-care facilities	Low
Batra, 2018, India^51^	South-East Asia	Pilot	Primary care setting	Low
Burgette, 2018, USA^52^	Americas	Retrospective	Primary care setting	Moderate
George, 2018, Australia^53^	Western Pacific	Randomised controlled trial	Primary care setting	High
Janssens, 2018, Belgium^54^	Europe	Non-randomised controlled trial	Nursing homes	Moderate
Lambert, 2018, Belgium^55^	Europe	Cross-sectional	Community	Moderate
Nelson, 2018, USA^56^	Americas	Qualitative research	Primary care setting	High
Simon, 2018, USA^57^	Americas	Retrospective	Community	Moderate
Trudnak Fowler, 2018, USA^58^	Americas	Mixed-method (policy analysis)	School	Moderate
Tynan, 2018, Australia^59^	Western Pacific	Mixed-method	Aged-care facilities	Moderate
Ajwani, 2019, Australia^60^	Western Pacific	Qualitative research	Primary care setting	High
Basso, 2019, Brazil^61^	Americas	Qualitative research	Primary care setting	Low
Dahlen, 2019, Australia^62^	Western Pacific	Qualitative research	Primary care setting	High
George, 2019, Australia^63^	Western Pacific	Qualitative research	Primary care setting	High
Villena, 2019, Peru^64^	Americas	Randomised controlled trial	Primary care setting	Moderate
Aagaard, 2020, Denmark^65^	Europe	Qualitative research	Nursing homes	High
Aronoff-Spencer, 2020, USA^66^	Americas	Pilot	Community	Moderate
Kanan, 2020, USA^67^	Americas	Pre–post intervention	Primary care setting	High
Song, 2020, USA^68^	Americas	Retrospective	Primary care setting	Moderate
Wood, 2020, USA^69^	Americas	Qualitative research	Primary care setting	High
Arif, 2021, USA^70^	Americas	Pre–post intervention	Primary care setting	High
Brännemo, 2021, Sweden^71^	Europe	Non-randomised controlled trial	Community	Low
Ho, 2021, Netherlands^72^	Europe	Mixed-method	Community	High
Pawloski, 2022, USA^73^	Americas	Qualitative research	Primary care setting	High

*Primary care settings include federally qualified health centres; accountable care organisations; nurse-managed health centres; prenatal/antenatal/maternal care; women, infants and children programme sites; antenatal care in hospitals; immunisation visits.

Table 2 presents the integration strategies and their alignment with WHO’s HSBB. In terms of integration strategies, most studies explored the provision of oral healthcare by non-dental primary care professionals in the primary care setting (n=24), where integration was achieved primarily via training/education and/or policy changes. Some strategies were implemented in the community setting and focused on interprofessional collaboration between community health workers and oral health professionals,^31 33 48^ delivery of community-based oral health services by dental, medical and allied health students,^30 57^ and oral health promotion by trained community nutrition educators.^27^ Other strategies involved policy changes including the extension of scope for non-dental primary care professionals (general practitioners (GPs), midwives, nurses) to cover oral health with support for the required training,^52^ expansion of health insurance policy coverage to include oral health and reimburse non-dental primary care professionals for oral health services,^38^ extend eligibility to subgroups such as people with a disability,^68^ and organisational changes to facilitate integration.^29^ Studies on colocation of dental and medical services,^25 69^ school-based preventive oral hygiene activities for children,^46^ use of teledentistry for providing oral healthcare^59^ and use of an integrated oral health assessment tool^66^ were also reviewed.

**Table 2:** Integration strategies and alignment with WHO’s Health System Building Blocks

Study	Integration strategy	Health system building blocks					
		SD	HW	HIS	Med	Fin	Gov
Mason *et al* 1994^25^	Colocation of medical and dental practices	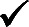		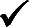			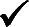
O'Neil and Clarkson 2002^26^	Physicians providing oral healthcare	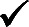	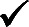				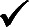
Lawrence *et al* 2004^27^	Community-based oral health promotion programme	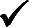	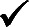			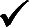	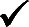
Fallon *et al* 2006^28^	Oral healthcare training for facility staff	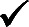	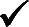			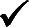	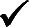
Niiranen *et al* 2008^29^	Policy change: Oral healthcare provided based on dental indications of need	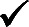	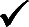			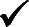	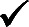
Mofidi and Gambrell 2009^30^	Dental students and residents in a community-based setting	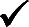	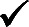			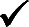	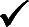
Silk *et al* 2010^31^	Community (including other health professionals) and dental staff collaboration	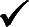	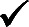			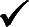	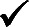
Skapetis *et al* 2012^32^	Training for emergency department staff—‘Management of Dental Emergencies’	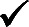	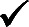				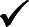
Vichayanrat *et al* 2012^33^	Primary care professionals and community members supporting oral healthcare	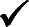	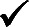				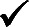
McKeown *et al* 2014^34^	Oral healthcare training for facility staff	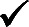	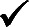			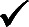	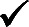
Biordi *et al* 2015^35^	Primary care professionals providing oral healthcare	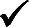	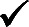		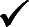		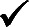
De Visschere *et al* 2015^36^	Oral healthcare training for nursing staff	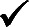	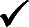				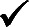
Heilbrunn-Lang *et al* 2015^37^	Midwives providing oral healthcare	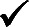	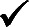			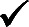	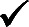
Kranz *et al* 2015^38^	Policy change: Reimbursement to primary care physicians for oral healthcare	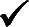	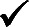			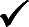	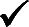
McNally *et al* 2015^39^	Oral healthcare training for facility staff	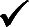	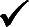				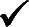
Crall *et al* 2016^40^	Primary care professionals providing oral healthcare	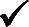	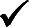		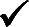	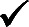	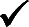
de Mey *et al* 2016^41^	Training and supervision of mental health nursing staff	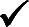	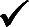				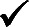
Dooley *et al* 2016^42^	Primary care professionals providing oral healthcare	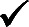	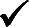		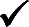	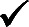	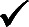
George *et al* 2016^43^	Oral healthcare training for midwives	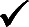	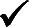				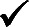
Vece *et al* 2016^44^	Primary care professionals providing oral healthcare	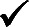	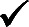				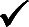
Adams *et al* 2017^45^	Oral health education and oral hygiene skills module delivered by nurse-midwives	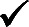	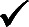				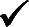
Duijster *et al* 2017^46^	Daily practice of group hygiene activities (hand washing and tooth brushing) and school-based deworming	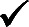	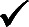		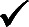	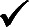	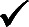
Kohli *et al* 2017^47^	Oral healthcare training for facility staff	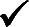	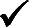			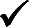	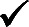
Mathu-Muju *et al* 2017^48^	Community health workers collaborate with dental therapists and dental hygienists	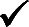	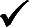			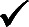	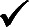
Sengupta *et al* 2017^49^	Primary care professionals providing oral healthcare	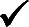	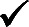	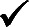		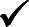	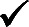
Wright *et al* 2017^50^	Training for staff, assistance with preparing individualised daily oral healthcare plans and onsite dental services	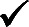	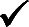			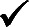	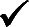
Batra *et al* 2018^51^	Community health workers providing oral healthcare	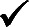	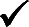				
Burgette *et al* 2018^52^	Policy change: educate all Early Head Start programme staff about oral health	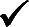	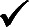				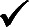
George *et al* 2018^53^	Midwives providing oral healthcare	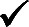	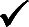				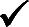
Janssens *et al* 2018^54^	Oral healthcare programme	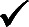	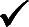				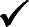
Lambert 2018^55^	Oral healthcare training for community volunteers	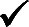	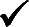				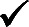
Nelson *et al* 2018^56^	Primary care professionals providing oral healthcare	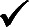	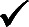			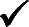	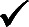
Simon *et al* 2018^57^	Dental, medical and nursing students delivering community-based oral healthcare	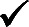	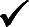				
Trudnak Fowler *et al* 2018^58^	Oral health services integrated within existing school-based health centres	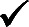				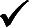	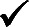
Tynan *et al* 2018^59^	Telehealth: Remote real-time oral examination	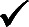	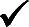		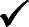		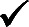
Ajwani *et al* 2019^60^	Midwives providing oral healthcare	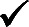	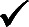				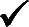
Basso *et al* 2019^61^	An oral health team is organised to work together with a family health team	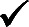	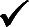	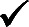		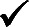	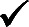
Dahlen *et al* 2019^62^	Midwives providing oral healthcare	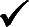	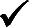				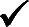
George *et al* 2019^63^	Midwives providing oral healthcare	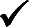	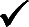				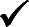
Villena *et al* 2019^64^	Training for nurses to deliver oral healthcare	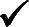	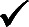		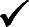		
Aagaard *et al* 2020^65^	Training and education for nursing staff	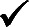	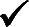			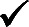	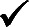
Aronoff-Spencer *et al* 2020^66^	Geriatric Assessment tool with an oral health assessment and a real-time referral process	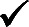			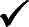		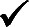
Kanan *et al* 2020^67^	Oral healthcare training for primary care professionals	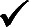	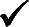	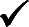			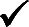
Song *et al* 2020^68^	Policy change: Access to Medicaid managed care plans for children with disabilities	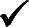	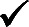		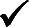	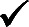	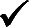
Wood *et al* 2020^69^	Colocation and collaboration between oral and medical services	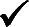	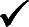	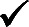			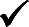
Arif *et al* 2021^70^	Oral healthcare training for primary care professionals	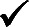	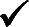			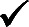	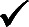
Brännemo *et al* 2021^71^	Child health nurses providing oral health education	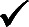	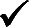		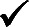		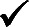
Ho *et al* 2021^72^	Oral healthcare training for primary care professionals and home care workers	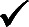	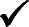	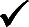			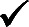
Pawloski *et al* 2022^73^	Oral health professionals provide oral health education and preventive care during primary healthcare visits	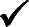	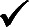		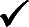		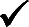

Fin, financing; Gov, governance and leadership; HIS, health information systems; HW, health workforce; Med, access to essential medicines and technology; SD, service delivery.

Most integration strategies, shown in table 2, aligned with the ‘Service delivery’, ‘Health workforce’ and ‘Governance’ HSBBs. In order to achieve integration, interventions were focused on enhancing the competencies and reorienting the responsibilities of healthcare professionals, often involving organisational-level actions. Few integration strategies corresponded to the ‘Financing’, ‘Access to medical products/technologies’ and ‘Health information systems’ HSBBs. An example of a strategy intersecting with the ‘Financing’ HSBB was policy reform to reimburse primary care physicians who performed oral care procedures,^38^ while use of teledentistry to aid in oral healthcare provision to remote communities best illustrates the ‘Medical products/technology’ component.^59^

**Figure 3:**
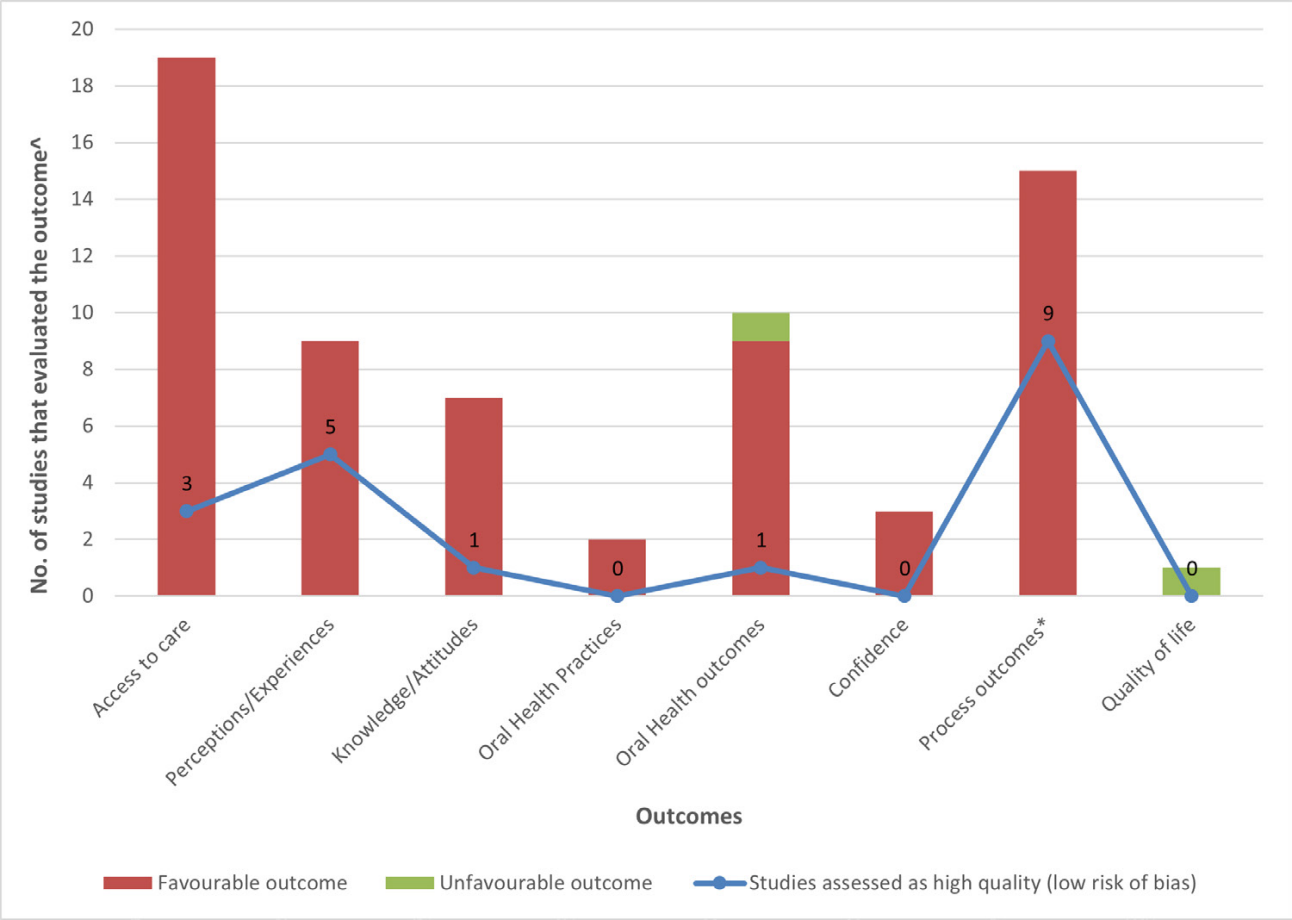
Summary of outcomes and key findings of included studies. ˆSome included studies evaluated more than one outcome and are included in each of the outcome. Therefore, the overall total will be more than 49; *Process outcomes include implementation barriers and facilitators; all high-quality studies reported favourable outcome.

Figure 3 illustrates the summary of outcomes of interest and key results of the included studies (details presented in online supplemental table S5). Access to oral healthcare was the primary outcome most studied (n=19), followed by knowledge/attitudes/perceptions of staff and patients (n=16). Nine studies reported on oral disease outcomes, seven of which measured change in dental caries estimates.^33 35 38 45 46 64 71^ Overall, most studies reported results favouring the integration strategy, except two,^33 59^ that reported no difference in the outcomes measured. The success of integration strategies in improving access to oral healthcare included improvements in referral pathways, documentation processes, operating efficiencies, the number of available health staff, increased number of visits for oral health issues, increased proportion of children receiving fluoride varnish applications/other preventive treatment, and increased proportion of visits to an oral health professional. For example, by integrating oral health into primary care at Federally Qualified Health Centres, Crall *et al* 40 demonstrated a threefold increase (from 3000 to 10 000 children over 2 years) in the number of children that received preventive treatment (including fluoride varnish applications). The most commonly studied qualitative outcomes were barriers and facilitators related to the strategy implementation process. Barriers reported were time and staff constraints, resource limitations and lack of organisational support. Facilitators were knowledge and attitude of healthcare professionals. Patient and staff perceptions on various aspects of the integration strategy were usually reported as positive in the included studies.

## Discussion

This systematic review collated the available information in the published peer-reviewed literature on strategies to integrate oral health into primary care. The information included (but was not limited to) study design, integration strategies, outcomes measured, facilitators/barriers to implementation of the integration strategy, study location and effectiveness. Of the 49 studies included, only 2 were RCTs.^53 64^ Facilitating an integrated approach for oral health is a complex process which is often impeded by health system-level barriers, professional training/education and professional turf boundaries.^12^ In such circumstances, opportunities for research itself may be limited and any form of a research trial which requires complex research methodology, long-term commitment and resourcing would be a rarity. For the reasons stated in the previous sentences, it was not a surprising finding that (A) only two RCTs were reported and (B) the majority of the research on this topic was conducted in countries classified as high-income. Similar findings regarding study design and setting (high-income countries) were also identified in two recent reviews on this topic.^14 74^ Several novel aspects that were the focus of this systematic review (compared with previous reviews on this topic) were the primary care setting, strategies that were evaluated, outcomes that were directly related to practice or patient care, the effectiveness of the integration strategy and using WHO’s HSBB to provide a systems perspective to the analysis.

Most strategies identified in this review, utilised non-dental primary care professionals in the primary care setting. This finding was similar to the integration strategies identified in a recent review on the same topic. However, the review by Prasad *et al* 14 was a broad scoping exercise which included integration strategies in the dental setting and within oral health professionals; and did not study outcomes or the success of the integration strategy.^14^ The fact that most strategies used non-dental primary care professionals like GPs, nurses and midwives is not surprising as this workforce have more frequent interactions and a trusting relationship with patients thus putting them in a unique position to promote oral health.^75^ Further, studies have shown that with proper oral health training and resources non-dental health professionals are very receptive to playing an active role in this area.^76 77^ It is equally important that oral health training is incorporated into undergraduate curricula to ensure new graduates across the broader workforce are ready to incorporate oral health into their practice.^78^ These strategies can improve oral health awareness in the community which can have a stronger influence on whether people access dental care compared with having affordable dental referral pathways.

As a key focus of this review was on integration strategies that were operational, it was not surprising that the most frequently reported outcome was access to services. Access to care and UHC are key themes in WHO’s Framework on Integrated People-Centred Health Services.^9^ The vision set out in this framework is ‘all people have equal access to quality health services that are coproduced in a way that meets their life course needs, are coordinated across the continuum of care, and are comprehensive, safe, effective, timely, efficient and acceptable; and all carers are motivated, skilled and operate in a supportive environment’.^9^ It is about people receiving the right care, at the right time, by the right professional and in the right place. A number of studies in this review also reported on evaluation outcomes from the implementation process, such as staff and/or patient experiences and/or satisfaction^26 32 37 43 48 54 60 62 63 65 69 70 73^ and facilitators and barriers to implementation.^27 28 33 36 39 41 44 47 51 56 58 59 70 72 73^ Harnagea *et al*, in their recent scoping review, provide a detailed report on the barriers and facilitators in the integration of oral healthcare into primary care,^12^ which are similar to the findings in this review and as such will not be discussed in detail. Again, often reported facilitators were related to governance (including integration policy and leadership) and resourcing. The findings in this review and the scoping review by Harnagea *et al*,^12^ complement and reinforces the previously discussed findings by Mulvale *et al*,^74^ that formal governance interventions and structures play an important role in integration strategies. Health systems are recognised, globally, as being complex, dynamic and deeply rooted in the political context/climate of where they are located.^79 80^ As such, to reorient existing systems towards integration is a lengthy process that requires action (including political), leadership and change management at every level of the health system. Hence, it was not surprising that only nine studies^33 35 38 41 45 46 50 64 71^ in this review reported oral disease outcomes—the long-term goal or a measure of impact of an integration strategy.

For this systematic review, any change in the reported outcome, due to the integration strategy, was considered a measure of effectiveness. Majority of the integration strategies that aimed to improve access to care were successful in achieving this outcome. These included health insurance policy changes (expand population scope or reimbursement);^29 38 52 68^ health programmes and research studies where non-dental primary care professionals (GPs, nurses and midwives) provided oral healthcare;^26 31 33 35 37 40 42 44 49 51 56 60 62 63 71^ and colocation of services.^25 69^ Access to healthcare is considered a proxy measure for health outcomes, however, the meaning attached to the term ‘access’ can vary depending on context and study/programme purpose.^81^ Two basic concepts in relation to access to care that should be understood and recognised are ‘having access’ and ‘gaining access’. ‘Having access’ is about the potential to use a service and ‘gaining access’ is about the actual utilisation of a service.^81^ It is important to differentiate between these two concepts when studying integration strategies as it provides insight into where the strategy (study/programme/policy) is in terms of its implementation process. Among studies aligned with ‘having access’ were integration strategies that improved referral pathways, documentation processes, operating efficiencies and the number of available health staff.^25 49 50 57 61 66 72^ ‘Having access’ is not always associated with actual utilisation of a service; a fact that was highlighted in the recent Victorian Auditor-General’s Report on access to public dental services in Victoria, Australia, which reported that only one in four eligible people actually use the service.^82^ Hence, while strategies aligned with the ‘having access’ concept can potentially increase the utilisation of healthcare, whether they actually do requires further investigation.

The ‘gaining access’ concept, as stated earlier, is related to actual service utilisation. In fact, some scholars on the topic of access to healthcare have argued that utilisation of a service (and not potential to use) is the only true measure of access to care.^83^ This does not mean that strategies aligned with the ‘having access’ concept are not useful—it may be that these strategies are early in their development and evaluation phase, and as such have not yet been able to measure service utilisation. An interesting finding in this review was that all of the health insurance policy-related strategies were aligned with the ‘gaining access’ concept and reported improved utilisation of services for oral healthcare in the primary care setting,^29 52 68^ such as increases in the number of visits for oral health issues, the proportion of children receiving fluoride varnish applications/other preventive treatment, and the proportion of visits to an oral health professional. The success of integration strategies that are based on macro level policy in improving utilisation of services is not surprising as some of the largest public health gains have been the result of policy action.^84^ However, policy action and evaluation can be a complex systemic process very much determined by the political climate of the time. Freund *et al*,^85^ in their review on policy evaluation, report three main barriers to robust policy evaluation: political environment; lack of investment in evaluation capacity as part of policy implementation; and academic researcher’s lack of understanding of the complex political environment in which policies are developed and implemented, which results in their inability to undertake meaningful and robust evaluation of public health policy. These barriers could be potential reasons for why only five of the identified strategies in this review were related to policy action.

Only nine studies in this review reported oral disease outcomes as part of the evaluation of the integration strategy.^33 35 38 41 45 46 50 64 71^ Eight studies^35 38 41 45 46 50 64 71^ reported improved oral disease outcomes while one^33^ reported no change in oral disease status between the intervention and control groups. The primary reason for the study by Vichayanrat *et al*,^33^ being unable to demonstrate an improvement in oral disease (dental caries) outcomes was most likely due to the short follow-up time of 1 year. It is well documented that dental caries is a slow progressing disease which requires several years for disease development and progression.^86^

A clear observation from this review was that the majority of identified integration strategies were aligned with at least four HSBBs—Service delivery, Health workforce, Finance and Governance. This observation alludes to the complexity of the integration process and highlights the various components of the health system that needs to be navigated in order to achieve integration. Successful integration of oral health services requires a high degree of ‘goodness of fit’ between the current health system, its current HSBBs, and the envisioned service to be integrated. Health systems need to be ready to undertake integration, and therefore, will need strengthening of specific building blocks to accommodate the integration process and outcomes. For example, this systematic review has demonstrated that policies for integration need to be in place (governance), benefit packages and financial incentives considered (financing), training and task shifting undertaken (workforce), information systems aligned and clinical guidelines updated (service delivery), to achieve improved individual and health system outcomes.

We can confidently recommend, based on the findings of this systematic review, that non-dental primary care professionals are well placed to improve access to oral healthcare through several mechanisms. It is important that government organisations implement supportive policy changes to incorporate oral health into primary care as a first step to initiate culture change among non-dental primary care providers. These policies need to be flexible and consider staff constraints and resource limitations frequently encountered across primary care settings. Equally important is the need to provide oral health training through professional development activities to improve the knowledge and attitude of the various primary care providers and define their scope of practice in this area. Further, the inclusion of oral health into the undergraduate curriculum of key disciplines like nursing/midwifery, pharmacy and general medicine could help facilitate sustainable change regarding oral healthcare from the grassroots level. Policy-makers should also consider the cost–benefits of incentivising primary care providers to promote oral health. Capacity building of non-dental health professionals in this area would be invaluable in countries that lack well-developed oral health services as well as in rural/remote parts of many countries where access to oral health professionals is an issue.^87^ Lastly, the use of technology to enable oral healthcare in primary care settings should be promoted to support non-dental healthcare providers, particularly during COVID-19 and in regions that have limited access to affordable and accessible dental services.

There are several limitations that should be considered in interpreting the results of this study. First, the majority of included studies were low to moderate quality, with only 16 of the 49 included studies being assessed as high quality. However, the Cochrane Health Promotion and Public Health Field acknowledges the difficulty in assessing the quality of public health and health promotion studies.^88^ This is due to both the wide variety of designs used for these studies, and because techniques such as blinding are not always feasible. In addition, most current quality assessment (risk of bias) tools are directed towards studies with experimental study designs. As a result, current recommendations are to avoid weighting evidence from included studies according to study quality, and this was ensured in this review.^88^ Although a comprehensive search strategy was used to ensure identification of relevant articles, reporting bias may have an influence on the number of identified studies that did not show evidence of effectiveness. In addition, there was significant heterogeneity between included studies, including differences in integration strategies, settings, study designs and outcomes assessed, limiting comparability of findings. As a result, meta-analysis was not possible for this review, and thus outcomes could not be quantitatively compared. Furthermore, the majority of included studies were from the USA, Canada and Australia, and as only publications in the English language were included, the generalisability of findings to non-Western and low-income countries may be limited.

While this review provides an evidence base to inform country action, it is clear that integration of oral health into primary care is an emerging area of interest, where more research and evaluation is required to identify best practice models of service integration and provide the evidence to inform policy. A major research gap was the lack of studies that evaluated the economics of integration, without which it will be a challenge to convince policy-makers and decision influencers to support integration strategies. Furthermore, literature from low-middle-income countries was lacking, which is understandable (but not acceptable) given limitations in financial and technical support for strengthening advocacy, leadership, political commitment and delivery of integrated oral healthcare.^12^

This systematic review collated current literature and provided evidence for the effectiveness of strategies to integrate oral health into primary care. The complexity of the topic is reflected in the review itself which identified various strategies, settings, outcomes and populations. A common theme across the various integration strategies was the provision of oral healthcare in the primary care setting by non-dental primary care professionals (health workforce), with governance and financing interventions being key drivers of the integration strategy. Future studies on this topic must employ rigorous research methodology and the focus should be on service quality and utilisation, the role of technology, disease outcomes, low-middle-income countries/rural-remote-regional areas, policy analysis and health economics.

## Data Availability

All data relevant to the study are included in the article or uploaded as online supplemental information.
