# Supplementary material for: Strategies to integrate oral health into primary care: a systematic review
Source: BMJ Open. 2023 Jul 5;13(7):e070622. doi: 10.1136/bmjopen-2022-070622 (PMC10367016; doi:10.1136/bmjopen-2022-070622)
Supplement: Supplementary data [file bmjopen-2022-070622supp001.pdf]

Supplementary file

Table S1: Search terms based on the four concepts identified within the review question

Concept: Oral health	*Concept: Primary health*	*Concept: Strategies*	*Concept: Integration*
- Oral health (MeSH)<br>- Oral hygiene (MeSH)<br>- oral health<br>- oral hygiene<br>- oral care<br>- Dental caries (MeSH)<br>- Dental care (MeSH)<br>- dental health<br>- dental caries<br>- dental hygiene<br>- dental care<br>- dental hygienist<br>- Oral health service<br>- Dental health service<br>- Dental health care<br>- Dental education<br>- oral health education	- Primary health<br>- Primary health care [MeSH]<br>- Health care<br>- General health<br>- General practice<br>- Community health services<br>- Allied health<br>- Primary care nursing	- Implement*<br>- Strateg*<br>- Approach*<br>- Intervention*<br>- Implementation Strateg*<br>- Implementation<br>- Promot*<br>- Improv*<br>- Guideline*<br>- Recommend*<br>- Consensus<br>- Disseminat*<br>- Translat*<br>- Guideline implementation<br>- Clinical guideline<br>- Clinical protocols<br>- Polic*<br>- Screening<br>- Protocol<br>- Health plan implementation [MeSH]	- Integrat*<br>- Inter disciplinary<br>- Inter professional<br>- Multi disciplinary<br>- Multi professional<br>- Collaborat*<br>- Collaborative practice<br>- Coordination*<br>- Co ordination*

Table S2: Detailed search strategies for all electronic databases

**MEDLINE (via OVID) –** no limit to 20 June 2022		
**Search ID#**	**Search Terms**	**Results**
1	oral health.mp. [mp=title, abstract, original title, name of substance word, subject heading word, floating sub-heading word, keyword heading word, organism supplementary concept word, protocol supplementary concept word, rare disease supplementary concept word, unique identifier, synonyms]	31856
2	oral hygiene.mp. [mp=title, abstract, original title, name of substance word, subject heading word, floating sub-heading word, keyword heading word, organism supplementary concept word, protocol supplementary concept word, rare disease supplementary concept word, unique identifier, synonyms]	21507
3	oral care.mp. [mp=title, abstract, original title, name of substance word, subject heading word, floating sub-heading word, keyword heading word, organism supplementary concept word, protocol supplementary concept word, rare disease supplementary concept word, unique identifier, synonyms]	2662
4	dental caries.mp. [mp=title, abstract, original title, name of substance word, subject heading word, floating sub-heading word, keyword heading word, organism supplementary concept word, protocol supplementary concept word, rare disease supplementary concept word, unique identifier, synonyms]	51120
5	dental care.mp. [mp=title, abstract, original title, name of substance word, subject heading word, floating sub-heading word, keyword heading word, organism supplementary concept word, protocol supplementary concept word, rare disease supplementary concept word, unique identifier, synonyms]	39898
6	dental health.mp. [mp=title, abstract, original title, name of substance word, subject heading word, floating sub-heading word, keyword heading word, organism supplementary concept word, protocol supplementary concept word, rare disease supplementary concept word, unique identifier, synonyms]	15269
7	dental hygiene.mp. [mp=title, abstract, original title, name of substance word, subject heading word, floating sub-heading word, keyword heading word, organism supplementary concept word, protocol supplementary concept word, rare disease supplementary concept word, unique identifier, synonyms]	3014
8	dental hygienist.mp. [mp=title, abstract, original title, name of substance word, subject heading word, floating sub-heading word, keyword heading word, organism supplementary concept word, protocol supplementary concept word, rare disease supplementary concept word, unique identifier, synonyms]	924
9	oral health service.mp. [mp=title, abstract, original title, name of substance word, subject heading word, floating sub-heading word, keyword heading word, organism supplementary concept word, protocol supplementary concept word, rare disease supplementary concept word, unique identifier, synonyms]	133
10	dental health service.mp. [mp=title, abstract, original title, name of substance word, subject heading word, floating sub-heading word, keyword heading word, organism supplementary concept word, protocol supplementary concept word, rare disease supplementary concept word, unique identifier, synonyms]	229
11	dental health care.mp. [mp=title, abstract, original title, name of substance word, subject heading word, floating sub-heading word, keyword heading word, organism supplementary concept word, protocol supplementary concept word, rare disease supplementary concept word, unique identifier, synonyms]	964
12	dental education.mp. [mp=title, abstract, original title, name of substance word, subject heading word, floating sub-heading word, keyword heading word, organism supplementary concept word, protocol supplementary concept word, rare disease supplementary concept word, unique identifier, synonyms]	5175
13	oral health education.mp. [mp=title, abstract, original title, name of substance word, subject heading word, floating sub-heading word, keyword heading word, organism supplementary concept word, protocol supplementary concept word, rare disease supplementary concept word, unique identifier, synonyms]	893
14	Oral Health/	16450
15	Oral Hygiene/	12853
16	Dental Caries/	45159
17	Dental Care/	21214
18	1 or 2 or 3 or 4 or 5 or 6 or 7 or 8 or 9 or 10 or 11 or 12 or 13 or 14 or 15 or 16 or 17	127149
19	primary health.mp. [mp=title, abstract, original title, name of substance word, subject heading word, floating sub-heading word, keyword heading word, organism supplementary concept word, protocol supplementary concept word, rare disease supplementary concept word, unique identifier, synonyms]	94015
20	health care.mp. [mp=title, abstract, original title, name of substance word, subject heading word, floating sub-heading word, keyword heading word, organism supplementary concept word, protocol supplementary concept word, rare disease supplementary concept word, unique identifier, synonyms]	791431
21	general health.mp. [mp=title, abstract, original title, name of substance word, subject heading word, floating sub-heading word, keyword heading word, organism supplementary concept word, protocol supplementary concept word, rare disease supplementary concept word, unique identifier, synonyms]	27058
22	general practice.mp. [mp=title, abstract, original title, name of substance word, subject heading word, floating sub-heading word, keyword heading word, organism supplementary concept word, protocol supplementary concept word, rare disease supplementary concept word, unique identifier, synonyms]	48104

23	community health services.mp. [mp=title, abstract, original title, name of substance word, subject heading word, floating sub-heading word, keyword heading word, organism supplementary concept word, protocol supplementary concept word, rare disease supplementary concept word, unique identifier, synonyms]	32291
24	allied health.mp. [mp=title, abstract, original title, name of substance word, subject heading word, floating sub-heading word, keyword heading word, organism supplementary concept word, protocol supplementary concept word, rare disease supplementary concept word, unique identifier, synonyms]	19518
25	primary care nursing.mp. [mp=title, abstract, original title, name of substance word, subject heading word, floating sub-heading word, keyword heading word, organism supplementary concept word, protocol supplementary concept word, rare disease supplementary concept word, unique identifier, synonyms]	657
26	Primary Health Care/	77278
27	19 or 20 or 21 or 22 or 23 or 24 or 25 or 26	889314
28	implement*.mp. [mp=title, abstract, original title, name of substance word, subject heading word, floating sub-heading word, keyword heading word, organism supplementary concept word, protocol supplementary concept word, rare disease supplementary concept word, unique identifier, synonyms]	493687
29	strateg*.mp. [mp=title, abstract, original title, name of substance word, subject heading word, floating sub-heading word, keyword heading word, organism supplementary concept word, protocol supplementary concept word, rare disease supplementary concept word, unique identifier, synonyms]	1096159
30	approach*.mp. [mp=title, abstract, original title, name of substance word, subject heading word, floating sub-heading word, keyword heading word, organism supplementary concept word, protocol supplementary concept word, rare disease supplementary concept word, unique identifier, synonyms]	1800916
31	intervention*.mp. [mp=title, abstract, original title, name of substance word, subject heading word, floating sub-heading word, keyword heading word, organism supplementary concept word, protocol supplementary concept word, rare disease supplementary concept word, unique identifier, synonyms]	1037026
32	implementation strateg*.mp. [mp=title, abstract, original title, name of substance word, subject heading word, floating sub-heading word, keyword heading word, organism supplementary concept word, protocol supplementary concept word, rare disease supplementary concept word, unique identifier, synonyms]	3635
33	implementation promot*.mp. [mp=title, abstract, original title, name of substance word, subject heading word, floating sub-heading word, keyword heading word, organism supplementary concept word, protocol supplementary concept word, rare disease supplementary concept word, unique identifier, synonyms]	21
34	improv*.mp. [mp=title, abstract, original title, name of substance word, subject heading word, floating sub-heading word, keyword heading word, organism supplementary concept word, protocol supplementary concept word, rare disease supplementary concept word, unique identifier, synonyms]	2505385
35	guideline*.mp. [mp=title, abstract, original title, name of substance word, subject heading word, floating sub-heading word, keyword heading word, organism supplementary concept word, protocol supplementary concept word, rare disease supplementary concept word, unique identifier, synonyms]	469423
36	recommend*.mp. [mp=title, abstract, original title, name of substance word, subject heading word, floating sub-heading word, keyword heading word, organism supplementary concept word, protocol supplementary concept word, rare disease supplementary concept word, unique identifier, synonyms]	660327
37	consensus.mp. [mp=title, abstract, original title, name of substance word, subject heading word, floating sub-heading word, keyword heading word, organism supplementary concept word, protocol supplementary concept word, rare disease supplementary concept word, unique identifier, synonyms]	178356
38	disseminat*.mp. [mp=title, abstract, original title, name of substance word, subject heading word, floating sub-heading word, keyword heading word, organism supplementary concept word, protocol supplementary concept word, rare disease supplementary concept word, unique identifier, synonyms]	149360
39	translat*.mp. [mp=title, abstract, original title, name of substance word, subject heading word, floating sub-heading word, keyword heading word, organism supplementary concept word, protocol supplementary concept word, rare disease supplementary concept word, unique identifier, synonyms]	360771
40	guideline implementation.mp. [mp=title, abstract, original title, name of substance word, subject heading word, floating sub-heading word, keyword heading word, organism supplementary concept word, protocol supplementary concept word, rare disease supplementary concept word, unique identifier, synonyms]	1301
41	clinical guideline.mp. [mp=title, abstract, original title, name of substance word, subject heading word, floating sub-heading word, keyword heading word, organism supplementary concept word, protocol supplementary concept word, rare disease supplementary concept word, unique identifier, synonyms]	1766
42	clinical protocols.mp. [mp=title, abstract, original title, name of substance word, subject heading word, floating sub-heading word, keyword heading word, organism supplementary concept word, protocol supplementary concept word, rare disease supplementary concept word, unique identifier, synonyms]	30188
43	polic*.mp. [mp=title, abstract, original title, name of substance word, subject heading word, floating sub-heading word, keyword heading word, organism supplementary concept word, protocol supplementary concept word, rare disease supplementary concept word, unique identifier, synonyms]	366361
44	screening.mp. [mp=title, abstract, original title, name of substance word, subject heading word, floating sub-heading word, keyword heading word, organism supplementary concept word, protocol supplementary concept word, rare disease supplementary concept word, unique identifier, synonyms]	604083

45	protocol.mp. [mp=title, abstract, original title, name of substance word, subject heading word, floating sub-heading word, keyword heading word, organism supplementary concept word, protocol supplementary concept word, rare disease supplementary concept word, unique identifier, synonyms]	334298
46	Health Plan Implementation/	5935
47	28 or 29 or 30 or 31 or 32 or 33 or 34 or 35 or 36 or 37 or 38 or 39 or 40 or 41 or 42 or 43 or 44 or 45 or 46	7188964
48	integrat*.mp. [mp=title, abstract, original title, name of substance word, subject heading word, floating sub-heading word, keyword heading word, organism supplementary concept word, protocol supplementary concept word, rare disease supplementary concept word, unique identifier, synonyms]	534097
49	inter disciplinary.mp. [mp=title, abstract, original title, name of substance word, subject heading word, floating sub-heading word, keyword heading word, organism supplementary concept word, protocol supplementary concept word, rare disease supplementary concept word, unique identifier, synonyms]	685
50	inter professional.mp. [mp=title, abstract, original title, name of substance word, subject heading word, floating sub-heading word, keyword heading word, organism supplementary concept word, protocol supplementary concept word, rare disease supplementary concept word, unique identifier, synonyms]	1466
51	multi disciplinary.mp. [mp=title, abstract, original title, name of substance word, subject heading word, floating sub-heading word, keyword heading word, organism supplementary concept word, protocol supplementary concept word, rare disease supplementary concept word, unique identifier, synonyms]	6471
52	multi professional.mp. [mp=title, abstract, original title, name of substance word, subject heading word, floating sub-heading word, keyword heading word, organism supplementary concept word, protocol supplementary concept word, rare disease supplementary concept word, unique identifier, synonyms]	1151
53	collaborat*.mp. [mp=title, abstract, original title, name of substance word, subject heading word, floating sub-heading word, keyword heading word, organism supplementary concept word, protocol supplementary concept word, rare disease supplementary concept word, unique identifier, synonyms]	148525
54	collaborative practice.mp. [mp=title, abstract, original title, name of substance word, subject heading word, floating sub-heading word, keyword heading word, organism supplementary concept word, protocol supplementary concept word, rare disease supplementary concept word, unique identifier, synonyms]	1546
55	co ordination*.mp. [mp=title, abstract, original title, name of substance word, subject heading word, floating sub-heading word, keyword heading word, organism supplementary concept word, protocol supplementary concept word, rare disease supplementary concept word, unique identifier, synonyms]	3604
56	coordination*.mp. [mp=title, abstract, original title, name of substance word, subject heading word, floating sub-heading word, keyword heading word, organism supplementary concept word, protocol supplementary concept word, rare disease supplementary concept word, unique identifier, synonyms]	115881
57	48 or 49 or 50 or 51 or 52 or 53 or 54 or 55 or 56	784016
58	18 and 27 and 47 and 57	981
59	limit 58 to dt=20200624-20220620	122

**Embase (via OVID)** – no limit to 24 June 2020		
**Search ID#**	**Search Terms**	**Results**
1	oral health.mp. [mp=title, abstract, heading word, drug trade name, original title, device manufacturer, drug manufacturer, device trade name, keyword, floating subheading word, candidate term word]	27301
2	oral hygiene.mp. [mp=title, abstract, heading word, drug trade name, original title, device manufacturer, drug manufacturer, device trade name, keyword, floating subheading word, candidate term word]	14555
3	oral care.mp. [mp=title, abstract, heading word, drug trade name, original title, device manufacturer, drug manufacturer, device trade name, keyword, floating subheading word, candidate term word]	3329
4	dental caries.mp. [mp=title, abstract, heading word, drug trade name, original title, device manufacturer, drug manufacturer, device trade name, keyword, floating subheading word, candidate term word]	60393
5	dental care.mp. [mp=title, abstract, heading word, drug trade name, original title, device manufacturer, drug manufacturer, device trade name, keyword, floating subheading word, candidate term word]	65218
6	dental health.mp. [mp=title, abstract, heading word, drug trade name, original title, device manufacturer, drug manufacturer, device trade name, keyword, floating subheading word, candidate term word]	16248
7	dental hygiene.mp. [mp=title, abstract, heading word, drug trade name, original title, device manufacturer, drug manufacturer, device trade name, keyword, floating subheading word, candidate term word]	3226
8	dental hygienist.mp. [mp=title, abstract, heading word, drug trade name, original title, device manufacturer, drug manufacturer, device trade name, keyword, floating subheading word, candidate term word]	1266
9	oral health service.mp. [mp=title, abstract, heading word, drug trade name, original title, device manufacturer, drug manufacturer, device trade name, keyword, floating subheading word, candidate term word]	122

10	dental health service.mp. [mp=title, abstract, heading word, drug trade name, original title, device manufacturer, drug manufacturer, device trade name, keyword, floating subheading word, candidate term word]	252
11	dental health care.mp. [mp=title, abstract, heading word, drug trade name, original title, device manufacturer, drug manufacturer, device trade name, keyword, floating subheading word, candidate term word]	1021
12	dental education.mp. [mp=title, abstract, heading word, drug trade name, original title, device manufacturer, drug manufacturer, device trade name, keyword, floating subheading word, candidate term word]	24095
13	oral health education.mp. [mp=title, abstract, heading word, drug trade name, original title, device manufacturer, drug manufacturer, device trade name, keyword, floating subheading word, candidate term word]	922
14	Oral Health/	180654
15	Oral Hygiene/	22983
16	Dental Caries/	57032
17	Dental Care/	65162
18	1 or 2 or 3 or 4 or 5 or 6 or 7 or 8 or 9 or 10 or 11 or 12 or 13 or 14 or 15 or 16 or 17	352338
19	primary health.mp. [mp=title, abstract, heading word, drug trade name, original title, device manufacturer, drug manufacturer, device trade name, keyword, floating subheading word, candidate term word]	81284
20	health care.mp. [mp=title, abstract, heading word, drug trade name, original title, device manufacturer, drug manufacturer, device trade name, keyword, floating subheading word, candidate term word]	1599994
21	general health.mp. [mp=title, abstract, heading word, drug trade name, original title, device manufacturer, drug manufacturer, device trade name, keyword, floating subheading word, candidate term word]	39448
22	general practice.mp. [mp=title, abstract, heading word, drug trade name, original title, device manufacturer, drug manufacturer, device trade name, keyword, floating subheading word, candidate term word]	101586
23	community health services.mp. [mp=title, abstract, heading word, drug trade name, original title, device manufacturer, drug manufacturer, device trade name, keyword, floating subheading word, candidate term word]	1354
24	allied health.mp. [mp=title, abstract, heading word, drug trade name, original title, device manufacturer, drug manufacturer, device trade name, keyword, floating subheading word, candidate term word]	11709
25	primary care nursing.mp. [mp=title, abstract, heading word, drug trade name, original title, device manufacturer, drug manufacturer, device trade name, keyword, floating subheading word, candidate term word]	221
26	Primary Health Care/	66086
27	19 or 20 or 21 or 22 or 23 or 24 or 25 or 26	1715673
28	implement*.mp. [mp=title, abstract, heading word, drug trade name, original title, device manufacturer, drug manufacturer, device trade name, keyword, floating subheading word, candidate term word]	648389
29	strateg*.mp. [mp=title, abstract, heading word, drug trade name, original title, device manufacturer, drug manufacturer, device trade name, keyword, floating subheading word, candidate term word]	1410276
30	approach*.mp. [mp=title, abstract, heading word, drug trade name, original title, device manufacturer, drug manufacturer, device trade name, keyword, floating subheading word, candidate term word]	2337061
31	intervention*.mp. [mp=title, abstract, heading word, drug trade name, original title, device manufacturer, drug manufacturer, device trade name, keyword, floating subheading word, candidate term word]	1467251
32	implementation strateg*.mp. [mp=title, abstract, heading word, drug trade name, original title, device manufacturer, drug manufacturer, device trade name, keyword, floating subheading word, candidate term word]	4397
33	implementation promot*.mp. [mp=title, abstract, heading word, drug trade name, original title, device manufacturer, drug manufacturer, device trade name, keyword, floating subheading word, candidate term word]	27
34	improv*.mp. [mp=title, abstract, heading word, drug trade name, original title, device manufacturer, drug manufacturer, device trade name, keyword, floating subheading word, candidate term word]	3553295
35	guideline*.mp. [mp=title, abstract, heading word, drug trade name, original title, device manufacturer, drug manufacturer, device trade name, keyword, floating subheading word, candidate term word]	755301
36	recommend*.mp. [mp=title, abstract, heading word, drug trade name, original title, device manufacturer, drug manufacturer, device trade name, keyword, floating subheading word, candidate term word]	990697
37	consensus.mp. [mp=title, abstract, heading word, drug trade name, original title, device manufacturer, drug manufacturer, device trade name, keyword, floating subheading word, candidate term word]	249049
38	disseminat*.mp. [mp=title, abstract, heading word, drug trade name, original title, device manufacturer, drug manufacturer, device trade name, keyword, floating subheading word, candidate term word]	209352
39	translat*.mp. [mp=title, abstract, heading word, drug trade name, original title, device manufacturer, drug manufacturer, device trade name, keyword, floating subheading word, candidate term word]	418759

40	guideline implementation.mp. [mp=title, abstract, heading word, drug trade name, original title, device manufacturer, drug manufacturer, device trade name, keyword, floating subheading word, candidate term word]	1851
41	clinical guideline.mp. [mp=title, abstract, heading word, drug trade name, original title, device manufacturer, drug manufacturer, device trade name, keyword, floating subheading word, candidate term word]	2774
42	clinical protocols.mp. [mp=title, abstract, heading word, drug trade name, original title, device manufacturer, drug manufacturer, device trade name, keyword, floating subheading word, candidate term word]	3973
43	polic*.mp. [mp=title, abstract, heading word, drug trade name, original title, device manufacturer, drug manufacturer, device trade name, keyword, floating subheading word, candidate term word]	513858
44	screening.mp. [mp=title, abstract, heading word, drug trade name, original title, device manufacturer, drug manufacturer, device trade name, keyword, floating subheading word, candidate term word]	1077607
45	protocol.mp. [mp=title, abstract, heading word, drug trade name, original title, device manufacturer, drug manufacturer, device trade name, keyword, floating subheading word, candidate term word]	552401
46	Health Plan Implementation/	97777
47	28 or 29 or 30 or 31 or 32 or 33 or 34 or 35 or 36 or 37 or 38 or 39 or 40 or 41 or 42 or 43 or 44 or 45 or 46	10026952
48	integrat*.mp. [mp=title, abstract, heading word, drug trade name, original title, device manufacturer, drug manufacturer, device trade name, keyword, floating subheading word, candidate term word]	655521
49	inter disciplinary.mp. [mp=title, abstract, heading word, drug trade name, original title, device manufacturer, drug manufacturer, device trade name, keyword, floating subheading word, candidate term word]	1137
50	inter professional.mp. [mp=title, abstract, heading word, drug trade name, original title, device manufacturer, drug manufacturer, device trade name, keyword, floating subheading word, candidate term word]	2562
51	multi disciplinary.mp. [mp=title, abstract, heading word, drug trade name, original title, device manufacturer, drug manufacturer, device trade name, keyword, floating subheading word, candidate term word]	14737
52	multi professional.mp. [mp=title, abstract, heading word, drug trade name, original title, device manufacturer, drug manufacturer, device trade name, keyword, floating subheading word, candidate term word]	2046
53	collaborat*.mp. [mp=title, abstract, heading word, drug trade name, original title, device manufacturer, drug manufacturer, device trade name, keyword, floating subheading word, candidate term word]	216844
54	collaborative practice.mp. [mp=title, abstract, heading word, drug trade name, original title, device manufacturer, drug manufacturer, device trade name, keyword, floating subheading word, candidate term word]	1972
55	co ordination*.mp. [mp=title, abstract, heading word, drug trade name, original title, device manufacturer, drug manufacturer, device trade name, keyword, floating subheading word, candidate term word]	5112
56	coordination*.mp. [mp=title, abstract, heading word, drug trade name, original title, device manufacturer, drug manufacturer, device trade name, keyword, floating subheading word, candidate term word]	134901
57	48 or 49 or 50 or 51 or 52 or 53 or 54 or 55 or 56	990609
58	18 and 27 and 47 and 57	5610

**CINAHL (vis EBSCOhost) -** no limit to 4 July 2020			
**Search ID#**	**Search Terms**	**Limiters/Expanders**	**Results**
1	S19 AND S29 AND S45 AND S55	Expanders - Apply equivalent subjects Search modes Boolean/Phrase	1,367
2	S46 OR S47 OR S48 OR S49 OR S50 OR S51 OR S52 OR S53 OR S54	Expanders - Apply equivalent subjects Search modes Boolean/Phrase	373,053
3	(MH "Interprofessional Relations+")	Expanders - Apply equivalent subjects Search modes Boolean/Phrase	32,037
4	(MH "Multidisciplinary Care Team+")	Expanders - Apply equivalent subjects Search modes Boolean/Phrase	48,459
5	coordinat*	Expanders - Apply equivalent subjects Search modes Boolean/Phrase	42,616
6	collaborat*	Expanders - Apply equivalent subjects Search modes Boolean/Phrase	114,532
7	multiprofessional	Expanders - Apply equivalent subjects Search modes Boolean/Phrase	1,225
8	multidisciplinary	Expanders - Apply equivalent subjects Search modes Boolean/Phrase	75,944
9	interprofessional	Expanders - Apply equivalent subjects Search modes Boolean/Phrase	33,336
10	interdisciplinary	Expanders - Apply equivalent subjects Search modes Boolean/Phrase	23,850
11	integrat*	Expanders - Apply equivalent subjects Search modes Boolean/Phrase	143,952

12	S30 OR S31 OR S32 OR S33 OR S34 OR S35 OR S36 OR S37 OR S38 OR S39 OR S40 OR S41 OR S42 OR S43 OR S44	Expanders - Apply equivalent subjects Search modes Boolean/Phrase	2,164,800
13	consensus	Expanders - Apply equivalent subjects Search modes Boolean/Phrase	46,325
14	guideline implementation	Expanders - Apply equivalent subjects Search modes Boolean/Phrase	4,433
15	screening	Expanders - Apply equivalent subjects Search modes Boolean/Phrase	180,241
16	clinical protocols	Expanders - Apply equivalent subjects Search modes Boolean/Phrase	11,597
17	polic*	Expanders - Apply equivalent subjects Search modes Boolean/Phrase	231,526
18	translat*	Expanders - Apply equivalent subjects Search modes Boolean/Phrase	60,405
19	disseminat*	Expanders - Apply equivalent subjects Search modes Boolean/Phrase	26,544
20	recommendation*	Expanders - Apply equivalent subjects Search modes Boolean/Phrase	121,446
21	guideline*	Expanders - Apply equivalent subjects Search modes Boolean/Phrase	207,923
22	improv*	Expanders - Apply equivalent subjects Search modes Boolean/Phrase	746,879
23	promot*	Expanders - Apply equivalent subjects Search modes Boolean/Phrase	245,259
24	intervention*	Expanders - Apply equivalent subjects Search modes Boolean/Phrase	496,823
25	approach*	Expanders - Apply equivalent subjects Search modes Boolean/Phrase	394,216
26	strateg*	Expanders - Apply equivalent subjects Search modes Boolean/Phrase	309,931
27	implement*	Expanders - Apply equivalent subjects Search modes Boolean/Phrase	220,487
28	S20 OR S21 OR S22 OR S23 OR S24 OR S25 OR S26 OR S27 OR S28	Expanders - Apply equivalent subjects Search modes Boolean/Phrase	1,060,598
29	(MH "Community Health Services+")	Expanders - Apply equivalent subjects Search modes Boolean/Phrase	450,369
30	(MH "Primary Health Care")	Expanders - Apply equivalent subjects Search modes Boolean/Phrase	66,921
31	primary care nursing	Expanders - Apply equivalent subjects Search modes Boolean/Phrase	2,062
32	allied health services	Expanders - Apply equivalent subjects Search modes Boolean/Phrase	417
33	community health services	Expanders - Apply equivalent subjects Search modes Boolean/Phrase	37,206
34	general practice	Expanders - Apply equivalent subjects Search modes Boolean/Phrase	36,624
35	general health care or general healthcare	Expanders - Apply equivalent subjects Search modes Boolean/Phrase	4,087
36	health care or healthcare	Expanders - Apply equivalent subjects Search modes Boolean/Phrase	682,364
37	primary health care or primary care or public health care	Expanders - Apply equivalent subjects Search modes Boolean/Phrase	141,153
38	S1 OR S2 OR S3 OR S4 OR S5 OR S6 OR S7 OR S8 OR S9 OR S10 OR S11 OR S12 OR S13 OR S14 OR S15 OR S16 OR S17 OR S18	Expanders - Apply equivalent subjects Search modes Boolean/Phrase	59,977
39	(MH "Dental Caries")	Limiters – English Language<br>Expanders - Apply equivalent subjects Search modes Boolean/Phrase	12,773
40	(MH "Dental Health Services+")	Limiters – English Language<br>Expanders - Apply equivalent subjects Search modes Boolean/Phrase	19,264
41	(MH "Dental Care+")	Limiters – English Language<br>Expanders - Apply equivalent subjects Search modes Boolean/Phrase	18,738
42	(MH "Oral Hygiene+")	Limiters – English Language<br>Expanders - Apply equivalent subjects Search modes Boolean/Phrase	7,756
43	(MH "Oral Health")	Limiters – English Language<br>Expanders - Apply equivalent subjects Search modes Boolean/Phrase	12,645
44	dental health education	Limiters – English Language<br>Expanders - Apply equivalent subjects Search modes Boolean/Phrase	1,275
45	dental care	Limiters – English Language<br>Expanders - Apply equivalent subjects Search modes Boolean/Phrase	23,477
46	dental health services	Limiters – English Language	1,918

		Expanders - Apply equivalent subjects Search modes Boolean/Phrase	
47	oral health education	Limiters – English Language<br>Expanders - Apply equivalent subjects Search modes Boolean/Phrase	1,335
48	oral health care	Limiters – English Language<br>Expanders - Apply equivalent subjects Search modes Boolean/Phrase	12,089
49	oral health services	Limiters – English Language<br>Expanders - Apply equivalent subjects Search modes Boolean/Phrase	882
50	dental caries	Limiters – English Language<br>Expanders – Apply equivalent subjects Search modes Boolean/Phrase	14,187
51	dental care	Limiters – English Language<br>Expanders - Apply equivalent subjects Search modes Boolean/Phrase	23,477
52	dental hygiene	Limiters – English Language<br>Expanders - Apply equivalent subjects Search modes Boolean/Phrase	18,042
53	dental health	Limiters – English Language<br>Expanders - Apply equivalent subjects Search modes Boolean/Phrase	8,322
54	oral care	Limiters – English Language<br>Expanders - Apply equivalent subjects Search modes Boolean/Phrase	14,637
55	oral hygiene	Limiters – English Language<br>Expanders - Apply equivalent subjects Search modes Boolean/Phrase	14,820
56	oral health	Limiters – English Language<br>Expanders - Apply equivalent subjects Search modes Boolean/Phrase	20,777

**ProQuest** – no limit to 5 July 2020		
**Search ID#**	**Search Terms**	**Results**
1	noft("oral health" OR "oral hygiene" OR "oral care" OR "dental health" OR "dental hygiene" OR "dental care" OR "dental caries" OR "oral health service" OR "dental health service" OR "oral health education" OR "dental education")	390400
2	noft("primary health" OR "primary health care" OR "primary healthcare" OR "health care" OR "healthcare" OR "general health care" OR "general healthcare" OR "general practice" OR "community health services" OR "allied health services" OR "primary care nursing")	11658946
3	noft("implement*" OR "strateg*" OR "approach*" OR "intervention*" OR "promot*" OR "improv*" OR "guideline*" OR "recommendation*" OR "disseminat*" OR "translat*" OR "polic*" OR "clinical protocols" OR "screening" OR "guideline implementation" OR "consensus")	89451823
4	noft("integrat*" OR "interdisciplinary" OR "interprofessional" OR "multidisciplinary" OR "multiprofessional" OR "collaborat*" OR "coordinat*")	12924323
5	noft("oral health" OR "oral hygiene" OR "oral care" OR "dental health" OR "dental hygiene" OR "dental care" OR "dental caries" OR "oral health service" OR "dental health service" OR "oral health education" OR "dental education") AND noft("primary health" OR "primary health care" OR "primary healthcare" OR "health care" OR "healthcare" OR "general health care" OR "general healthcare" OR "general practice" OR "community health services" OR "allied health services" OR "primary care nursing") AND noft("implement*" OR "strateg*" OR "approach*" OR "intervention*" OR "promot*" OR "improv*" OR "guideline*" OR "recommendation*" OR "disseminat*" OR "translat*" OR "polic*" OR "clinical protocols" OR "screening" OR "guideline implementation" OR "consensus") AND noft("integrat*" OR "interdisciplinary" OR "interprofessional" OR "multidisciplinary" OR "multiprofessional" OR "collaborat*" OR "coordinat*")	3301
6	noft("oral health" OR "oral hygiene" OR "oral care" OR "dental health" OR "dental hygiene" OR "dental care" OR "dental caries" OR "oral health service" OR "dental health service" OR "oral health education" OR "dental education") AND noft("primary health" OR "primary health care" OR "primary healthcare" OR "health care" OR "healthcare" OR "general health care" OR "general healthcare" OR "general practice" OR "community health services" OR "allied health services" OR "primary care nursing") AND noft("implement*" OR "strateg*" OR "approach*" OR "intervention*" OR "promot*" OR "improv*" OR "guideline*" OR "recommendation*" OR "disseminat*" OR "translat*" OR "polic*" OR "clinical protocols" OR "screening" OR "guideline implementation" OR "consensus") AND noft("integrat*" OR "interdisciplinary" OR "interprofessional" OR "multidisciplinary" OR "multiprofessional" OR "collaborat*" OR "coordinat*") AND la.exact("ENG")	3195

**Scopus** – no limit to 5 July 2020		
**Search ID#**	**Search Terms**	**Results**
1	TITLE-ABS-KEY ("oral health' OR "oral hygiene" OR "oral care" OR "dental health" OR 'dental hygiene' OR "dental care" OR "dental caries" OR "oral health service" OR "dental health service" OR oral health education' OR "dental education")	185210
2	TITLE-ABS-KEY ("primary health" OR "primary health care" OR "primary healthcare" OR "health care" OR"healthcare" OR "general health care" OR "general healthcare" OR "general practice" OR "community health services" OR "allied health services" OR "primary care nursing")	1990010
3	(TITLE-ABS-KEY ("implement*" OR "strateg*" OR "approach*" OR "intervention*" OR "promot*" OR "improv*" OR "guideline*" OR "recommendation*" OR "disseminat*" OR "translat*" OR "polic*" OR "clinical protocols" OR screening" OR "guideline implementation" OR "consensus"')	20585101
4	TITLE-ABS-KEY ("integrat*" OR "interdisciplinary' OR "interprofessional" OR "multidisciplinary" OR multiprofessional' OR "collaborat*" OR 'coordinat*")	4288165
5	(TITLE-ABS-KEY ("oral health" OR "oral hygiene" OR "oral care" OR 'dental health' OR "dental hygiene" OR "dental care" OR "dental caries" OR "oral health service" OR "dental health service" OR "oral health education" OR "dental education" AND (TITLE-ABS-KEY ("primary health" OR "primary health care" OR 'primary healthcare" OR "health care" OR "healthcare" OR "general health care" OR "general healthcare" OR "general practice" OR "community health services" OR "allied health services" OR "primary care nursing" AND (TITLE-ABS-KEY ("implement*" OR "strateg*" OR "approach*" OR "intervention*" OR •promot*" OR "improv*" OR "guideline*" OR recommendation*" OR "disseminat*" OR "translat*" OR "polic*" OR "clinical protocols" OR "screening" OR •guideline implementation" OR "consensus')) AND (TITLE-ABS-KEY("integrat*" OR "interdisciplinary" OR "interprofessional" OR "multidisciplinary" OR "multiprofessional" OR "collaborat*" OR "coordinat*"))	2126
6	(TITLE-ABS-KEY ("oral health" OR "oral hygiene" OR "oral care" OR 'dental health' OR "dental hygiene" OR "dental care" OR "dental caries" OR "oral health service" OR "dental health service" OR "oral health education" OR "dental education" AND (TITLE-ABS-KEY ("primary health" OR "primary health care" OR 'primary healthcare" OR "health care" OR "healthcare" OR "general health care" OR "general healthcare" OR "general practice" OR "community health services" OR "allied health services" OR "primary care nursing" AND (TITLE-ABS-KEY ("implement*" OR "strateg*" OR "approach*" OR "intervention*" OR •promot*" OR "improv*" OR "guideline*" OR recommendation*" OR "disseminat*" OR "translat*" OR "polic*" OR "clinical protocols" OR "screening" OR •guideline implementation" OR "consensus')) AND (TITLE-ABS-KEY("integrat*" OR "interdisciplinary" OR "interprofessional" OR "multidisciplinary" OR "multiprofessional" OR "collaborat*" OR "coordinat*")) AND (LIMIT LANGUAGE , "English"	2006

**Cochrane Library** – no limit to 5 July 2020		
**Search ID#**	**Search Terms**	**Results**
1	((“oral health” OR “oral hygiene” OR “oral care” OR “dental health” OR “dental hygiene” OR “dental care” OR “dental hygienist” OR “oral health service” OR “dental health service” OR “dental health care” OR “dental education” OR “oral health education”)):ti,ab,kw (Word variations have been searched)	7885
2	((“primary health” OR “primary health care” OR “health care” OR “general health” OR “general practice” OR “community health services” OR “allied heath services” OR “primary care nursing”)):ti,ab,kw (Word variations have been searched)	66385
3	((implement* OR strateg* OR approach* OR intervention* OR promot* OR improv* OR guideline* OR recommendation* OR consensus OR disseminat* OR translat* OR polic* OR screening OR protocol OR guideline implementation OR clinical protocols)):ti,ab,kw (Word variations have been searched)	804567
4	((integrat* OR interdisciplinary OR interprofessional OR multidisciplinary OR multiprofessional OR collaborat* OR coordination*)):ti,ab,kw (Word variations have been searched)	44574
5	MeSH descriptor: [Oral Health] this term only	393
6	MeSH descriptor: [Oral Hygiene] this term only	993
7	MeSH descriptor: [Dental Care] explode all trees	621
8	MeSH descriptor: [Dental Caries] this term only	2422
9	MeSH descriptor: [Primary Health Care] explode all trees	7095
10	MeSH descriptor: [Health Plan Implementation] explode all trees	170
11	#1 OR #5 OR #6 OR #7 OR #8	9644
12	#2 OR #9	68395
13	#3 OR #10	804567
14	#11 AND #12 AND #13 AND #4	114

**Google Scholar** – no limit to 5 July 2020		
**Search ID#**	**Search Terms**	**Results**
1	("oral health" OR "oral hygiene" OR "oral care" OR "dental health" OR "dental hygiene" OR "dental care" OR "dental caries" OR "oral health service" OR "dental health service" OR "oral health education" OR "dental education")	17,900
2	("primary health" OR "primary health care" OR "primary healthcare" OR "health care" OR "healthcare" OR "general health care" OR "general healthcare" OR "general practice" OR "community health services" OR "allied health services" OR "primary care nursing")	4,180,000
3	("implement*" OR "strateg*" OR "approach*" OR "intervention*" OR "promot*" OR "improv*" OR "guideline*" OR "recommendation*" OR "disseminat*" OR "translat*" OR "polic*" OR "clinical protocols" OR "screening" OR "guideline implementation" OR "consensus")	5,580,000
4	("integrat*" OR "interdisciplinary" OR "interprofessional" OR "multidisciplinary" OR "multiprofessional" OR "collaborat*" OR "coordinat*")	2,660,000
5	#1 AND #2 AND #3 AND #4	698
6	#5 AND Limit to English	540

Table S3: Inclusion and exclusion criteria

Criterion	Inclusion	Exclusion
Population	Any	None
Integration strategies	Any activity or combination of activities that aims to integrate oral health care/services into primary care. Such as: conceptual frameworks, guidelines, governance structures, policies, insurances, financial protections, and programs around (but not limited to) integration of care, health workforce, inter-professional practice, education and training.	Strategies that aim to integrate general/ primary care into the oral health setting - including education; Strategies in a non-primary care setting such as schools and community organisations that do not include other primary care professionals; Inter-professional education strategies that do not have a practice related outcome.
Outcomes	Including but not limited to fidelity, dose, reach and facilitators/barriers of the particular strategy, health service-related outcomes such as operating efficiencies (including cost), service staff related outcomes such as change in knowledge and attitudes; and patient related outcomes such as change in knowledge and attitudes and health related outcomes.	No direct practice/patient care related outcome.
Study design and information source	Only studies that included an evaluation or research component and which reported information on effectiveness/efficacy/success of the integration strategy.	Studies that described a strategy without an evaluation.
Publication type	Original peer-reviewed and published research.	Anything outside this parameter including, reports, newsletters, reviews, study protocol papers, oral or poster presentations and commentaries.
Language	English	Non-English

Table S4: Studies excluded at full-text stage with reasons

Study	Reason for exclusion
Otsuka et al, 2016[1]	Wrong outcomes
Haughney et al, 1998[2]	Full-text unavailable
Stevens et al, 2007[3]	No evaluation of the integration strategy
Hartnett et al, 2019[4]	Wrong outcomes
Niranjan et al, 2019[5]	Wrong outcomes
Dolce et al, 2018[6]	Wrong outcomes
Chinn, 2011[7]	Wrong outcomes
Braun et al, 2015[8]	Wrong outcomes
Clark et al, 2017[9]	Wrong outcomes
McConnell et al, 2007[10]	No evaluation of the integration strategy
Bassiouny et al, 2017[11]	Wrong publication type
Bonwell et al, 2014[12]	Wrong outcomes
Maxey et al, 2017[13]	No evaluation of the integration strategy
Murphy et al, 2017[14]	No evaluation of the integration strategy
Petrosky et al, 2009[15]	Wrong outcomes
Nair et al, 2009[16]	Wrong outcomes
Shrivastava et al, 2020[17]	No intervention/integration strategies
Haber, 2020[18]	Wrong outcomes
Gold et al, 2018[19]	No evaluation of the integration strategy
Mouradian et al, 2003[20]	Wrong outcomes
Mowat et al, 2017[21]	Wrong outcomes
Savageau et al, 2019[22]	Wrong outcomes
Nicolae et al, 2018[23]	No evaluation of the integration strategy
Scherr et al, 2020[24]	No evaluation of the integration strategy
Saparamadu, 1984[25]	No evaluation of the integration strategy
Wysen et al, 2004[26]	No evaluation of the integration strategy
Kimball et al, 2013[27]	No intervention/integration strategies
Anumanrajadhon et al, 1996[28]	No evaluation of the integration strategy
Markowski et al, 2018[29]	Wrong outcomes
Leone et al 2017[30]	Wrong outcomes
Lavigne, 1999[31]	Wrong outcomes
Clark, 2019[32]	No evaluation of the integration strategy
Janotha et al, 2019[33]	Wrong outcomes
Kordsmeier et al, 2020[34]	Wrong outcomes
Ivon Falcon et al, 2020[35]	Wrong outcomes
Nash et al, 2018[36]	Wrong outcomes
Berkowitz et al, 2017[37]	Wrong outcomes
Buck et al, 1999[38]	Wrong outcomes
King, 1984[39]	Wrong publication type
Lewis et al, 2013[40]	Wrong outcomes
Olatosi et al, 2019[41]	Wrong outcomes
Anderson et al, 2011[42]	Wrong outcomes
Thompson et al, 2016[43]	Wrong outcomes
Mouradian et al, 2006[44]	Wrong outcomes
Lary et al, 1997[45]	Wrong outcomes
Grant et al, 2011[46]	Wrong outcomes
Dolce et al, 2018[47]	Wrong outcomes
Karpa et al, 2019[48]	Wrong outcomes
Graham et al, 2003[49]	Wrong outcomes
Salama et al, 2010[50]	Wrong outcomes
Tong, 2015[51]	Wrong publication type
Jordan et al, 2012[52]	Non-English
Kobayashi et al, 2005[53]	No intervention/integration strategies
Macnab et al, 2008[54]	Wrong integration strategy
Martinez-Mier et al, 2011[55]	Wrong outcomes
Garland et al, 2014[56]	No evaluation of the integration strategy

Kendall, 2009[57]	Wrong integration strategy
More et al, 2005[58]	No evaluation of the integration strategy
Mayberry et al, 2020[59]	Wrong study design
Rothmund et al, 2017[60]	Wrong outcomes
Tynan et al, 2018[61]	Wrong integration strategy
Rowan et al, 2013[62]	Wrong integration strategy
Kaufman et al, 2016[63]	No intervention/integration strategies
McAnulla et al, 2018[64]	No evaluation of the integration strategy
Heuer et al, 2007[65]	No evaluation of the integration strategy
Jackson et al, 2015[66]	Wrong outcomes
Mann et al, 2017[67]	Not primary health care setting
Baker, 1990[68]	No evaluation of the integration strategy
Allen et al, 2004[69]	Wrong integration strategy
Manski et al, 2015[70]	No evaluation of the integration strategy
Mattevi et al, 2011[71]	Not primary health care setting
Dimmick et al, 2003[72]	No evaluation of the integration strategy
Kolisa et al, 2016[73]	No intervention/integration strategies
Charteris et al, 2001[74]	Not primary health care setting
Atchison et al, 2017[75]	No evaluation of the integration strategy
Jeganathan et al, 2010[76]	Not primary health care setting
Connor et al, 2007[77]	No evaluation of the integration strategy
Davis et al, 2010[78]	No intervention/integration strategies
Atchison et al, 2018[79]	No evaluation of the integration strategy
Atchison et al, 2018[80]	No evaluation of the integration strategy
Rabiei et al, 2016[81]	No intervention/integration strategies
Previato et al, 2018[82]	No intervention/integration strategies
Reeves et al, 1998[83]	No evaluation of the integration strategy
Mann et al, 2009[84]	No evaluation of the integration strategy
Myers-Wright et al, 2016[85]	No evaluation of the integration strategy
Merrick et al, 2000[86]	No evaluation of the integration strategy
Makuch et al, 2001[87]	Wrong integration strategy
Bowen 1995[88]	Wrong publication type
Alem et al, 2017[89]	Wrong publication type
Clarke, 2007[90]	Wrong publication type
Edwards, 2010[91]	Wrong publication type
Lapidos et al, 2016[92]	No evaluation of the integration strategy
Sullivan et al, 2016[93]	Wrong publication type
Taylor et al, 2011[94]	Wrong publication type
Vandamme et al, 2006[95]	Wrong integration strategy
Olatasi et al, 2020[96]	Wrong publication type
Collins et al, 2009[97]	No evaluation of the integration strategy
Dounis et al, 2013[98]	Wrong integration strategy
Duley et al, 2009[99]	Not primary health care setting
Dsouza et al, 2019[100]	Wrong integration strategy
Czarnecki et al, 2014[101]	Wrong outcomes
Cooper et al, 2017[102]	Wrong outcomes
Byrd et al, 2019[103]	Wrong outcomes
Boynes et al, 2017[104]	Wrong integration strategy
Bowser et al, 2013[105]	Wrong outcomes
Berg et al, 2012[106]	No evaluation of the integration strategy
Anders et al, 2016[107]	Wrong outcomes
Allen et al, 2019[108]	Wrong integration strategy
Nelson et al, 2015[109]	Wrong publication type
Meeks et al, 2016[110]	Wrong publication type
O' Reilly et al, 2019[111]	Wrong publication type
Khokhar et al, 2011[112]	Not primary health care setting
Forbes et al, 2018[113]	Wrong integration strategy
Golinveaux et al, 2013[114]	Wrong integration strategy

Haber et al, 2014[115]	No evaluation of the integration strategy
Haber et al, 2015[116]	Wrong publication type
Hahn et al, 2012[117]	Wrong integration strategy
Haresaku et al, 2020[118]	No evaluation of the integration strategy
Heath et al, 2019[119]	Wrong outcomes
Braun et al, 2016[120]	No evaluation of the integration strategy
Chung et al, 2020[121]	Wrong integration strategy
Biethman et al, 2017[122]	Wrong integration strategy
Beaglehole et al, 2019[123]	Wrong publication type
Larson et al, 2003[124]	No evaluation of the integration strategy
Stormon et al, 2018[125]	Not primary health care setting
Simon et al, 2017[126]	Wrong outcomes
NewsRx, 2019[127]	Wrong publication type
Shiel et al, 2012[128]	Wrong publication type
Mallonee, 2012[129]	Wrong publication type
Berge et al, 2013[130]	Wrong publication type
Giddon, 2018[131]	Wrong publication type
Marshall et al, 2013[132]	Wrong integration strategy
Norwood et al, 2017[133]	No intervention/integration strategies
Barsh, 2011[134]	Wrong publication type
Nishina et al, 2015[135]	Wrong outcomes
Donoff et al, 2014[136]	Wrong publication type
Bansal et al, 2019[137]	Not primary health care setting
Pucca et al, 2015[138]	Wrong integration strategy
Myers Virtue et al, 2018[139]	Wrong integration strategy
Janssen et al, 2007[140]	No intervention/integration strategies
McClure et al, 2017[141]	Wrong integration strategy
Kateeb et al, 2016[142]	No intervention/integration strategies
Jones et al, 2011[143]	Wrong outcomes
Laura et al, 2012[144]	Wrong integration strategy
Goyal et al, 2019[145]	Wrong study design
Taylor et al, 2014[146]	No evaluation of the integration strategy
Obana et al, 2019[147]	Not primary health care setting
Ho et al, 2005[148]	Wrong publication type
Rosedale et al, 2012[149]	Wrong integration strategy
Antonarakis, 2011[150]	No evaluation of the integration strategy
Lau et al, 2019[151]	No intervention/integration strategies
Vallabhan et al, 2020[152]	Wrong publication type
Crall et al, 2016[153]	Duplicate publication
Thomson et al, 2021[154]	No evaluation of the integration strategy
Niesten et al, 2021[155]	No intervention/integration strategies
Niesten et al, 2021[156]	No intervention/integration strategies
Pourat et al, 2020[157]	No intervention/integration strategies
Gesko et al, 2020[158]	No evaluation of the integration strategy
Dolce et al, 2020[159]	Not primary health care setting
Greiner et al, 2022[160]	No evaluation of the integration strategy
Tabrizi et al, 2020[161]	Wrong integration strategy
Pike et al, 2022[162]	Wrong integration strategy
Adeniyi et al, 2022[163]	No intervention/integration strategies
Ramos et al, 2020[164]	Wrong integration strategy
Linabarger et al, 2021[165]	Wrong integration strategy

Table S5. Summary of key findings of included studies

Study	Main outcome	Results
Mason et al (1994)[166]	Access to care	Improved referral pathways and efficiencies
O'Neil et al (2002)[167]	Perceptions	Physicians' perception that this was an effective way to reach families
Lawrence et al (2004)[168]	Knowledge; Oral health practices	Improved caregivers' knowledge, attitudes and practices in relation to their infant's oral health
Fallon et al (2006)[169]	Knowledge; Process outcomes	Improved mean knowledge score compared to the pre-implementation; Implementation barriers identified
Niiranen et al (2008)[170]	Access to care	Improved use of public dental services in the study area
Mofidi et al (2009)[171]	Access to care	Increase in the average number of visits to dental services
Silk et al (2010)[172]	Access to care	Number of dental visits doubled during the study period
Skapetis et al (2012)[173]	Knowledge; Confidence	Improvement in self-reported proficiency by all the participants
Vichayanrat et al (2012)[174]	Oral health outcomes; Oral health practices; Knowledge	No difference in decayed, missing, filled teeth (dmft) scores between groups; Increased use of fluoride products and knowledge in experimental group; Implementation barriers identified
McKeown et al (2014)[175]	Oral health outcomes	Modest reduction in oral debris prevalence; increase in the prevalence of inflammation
Biordi et al (2015)[176]	Oral health outcomes; Access to care	Reduction in mean caries estimate from first to second visit; improved access to dental services
De Visschere et al (2015)[177]	Process outcomes	Barriers and enablers to implementation were identified
Heilbrunn-Lang et al (2015)[178]	Confidence; Perceptions	Increase in confidence; perception that knowledge was relevant to practice and that oral health could be included in the first antenatal visit
Kranz et al (2015)[179]	Oral health outcomes	Lower caries estimates among children exposed to the intervention
McNally et al (2015)[180]	Process outcomes	Implementation barriers identified
Crall et al (2016)[181]	Access to care	Oral health visits increased after program implementation
de Mey et al (2016)[182]	Knowledge; Oral health outcomes	Improved staff knowledge and patient oral hygiene
Dooley et al (2016)[183]	Access to care	Increased proportion of children receiving fluoride varnish
George et al (2016)[184]	Knowledge; Perceptions; Confidence	Improved oral health knowledge and confidence of midwives. Training program was perceived to be useful for their practice.
Vece et al (2016)[185]	Process outcomes	All families found the oral health education helpful and were satisfied with the oral health care program
Adams et al (2017)[186]	Oral health outcomes	Significant improvements in plaque levels, bleeding on probing, pocket depth, and self-reported importance of oral health

Duijster et al (2017)[187]	Oral health outcomes	Children in intervention schools had lower dental caries estimates
Kohli et al (2017)[188]	Process outcomes	Participants agreed that they learned new skills and were competent to apply those new skills in daily care
Mathu-Muju et al (2017)[189]	Perceptions	Positive perceptions of all intervention areas
Sengupta et al (2017)[190]	Access to care	Increase in screening rates, referrals to the dental services, and fluoride varnish applications
Wright et al (2017)[191]	Oral health outcomes; Access to care	Increased proportion of residents' good oral hygiene; improved referral efficiencies
Batra et al (2018)[192]	Process outcomes	Social Health Activists' acceptability on integrating and delivering oral health with general health education
Burgette et al (2018)[193]	Access to care	Children in the program had greater odds of receiving a dental assessment and dental fluoride treatment
George et al (2018)[194]	Access to care	Intervention participants were significantly more likely to see a dentist during the pregnancy compared to control participants
Janssens et al (2018)[195]	Knowledge; Attitudes	There were significant improvements in knowledge and attitude in the intervention group
Lambert et al (2018)[196]	Access to care	Significant decrease in number of missed appointments and in the percentage of avoidable missed appointments
Nelson et al (2018)[197]	Process outcomes	Implementation barriers and facilitators identified
Simon et al (2018)[198]	Access to care	Improved documentation process
Trudnak Fowler et al (2018)[199]	Access to care; Process outcomes	Increased proportion who received preventive services, and completed treatments; factors contributing to the efficacy, implementation and sustainability of the strategy were identified
Tynan et al (2018)[200]	Quality of life; Process outcomes	No difference in quality of life scores. Implementation barriers and facilitators identified
Ajwani et al (2019)[201]	Perceptions & experiences	Positive perceptions of all program areas, including positive experiences from program participation; Implementation barriers and facilitators identified
Basso et al (2019)[202]	Access to care	Increase in the number of oral health teams and family health teams
Dahlen et al (2019)[203]	Process outcomes	Changed perceptions of the importance of oral health; program was acceptable and feasible; Implementation barriers identified
George et al (2019)[204]	Perceptions & experiences	Perceptions: the role of the midwife in oral health care was appropriate and satisfactory; the program increased their awareness about oral health; they had positive experiences of their involvement.
Villena et al (2019)[205]	Oral health outcomes	Lower caries estimates in the active intervention group
Aagaard et al (2020)[206]	Process outcomes	The intervention supported nursing staff to develop competencies in performing oral care
Aronoff-Spencer et al (2020)[207]	Access to care	Improved referral process

Kanan et al (2020)[208]	Process outcomes; Access to care	The initiative was effective in creating an operational structure for integrating oral health care into primary care practices. Increase in fluoride varnish applications and oral health assessments.
Song et al (2020)[209]	Access to care	More preventive visits by enrolled children
Wood et al (2020)[210]	Perceptions	Medical and dental professionals were able to leverage each other's expertise to effectively meet patients' health care needs
Arif et al (2021)[211]	Perceptions; Process outcomes; Access to care	Perceptions: Program participants felt comfortable with fluoride varnish applications, however, they were less comfortable performing oral screenings; Process: Implementation barriers and facilitators identified; Access: increase in preventive oral health services in the children
Brannemo et al (2021)[212]	Oral health outcomes	Reduction in the proportion of children with caries lesions. Improvement in oral hygiene habits in the intervention group.
Ho et al (2021)[213]	Process outcomes	Initiative was implemented, delivered and received as planned at macro, meso and micro levels; Improved collaborations between the general practices, dental practices, and home care organizations; Implementation barriers and facilitators identified
Pawloski et al (2022)[214]	Perceptions; Process outcomes	The program was perceived as beneficial and successful; Implementation barriers and facilitators identified

Note: Grey highlight = Studies assessed as high quality (low risk of bias)

References:

1. Otsuka H, Kondo K, Ohara Y, et al. An Inter- and Intraprofessional Education Program in Which Dental Hygiene Students Instruct Medical and Dental Students. *J Dent Educ* 2016; **80**(9): 1062-70.
2. Haughney MGJ, Devennie JC, Macpherson LMD, Mason DK. Integration of primary care dental and medical services: A three-year study. *Br Dent J* 1998; **184**(7): 343-7.
3. Stevens J, Iida H, Ingersoll G. Implementing an oral health program in a group prenatal practice. *J Obstet Gynecol Neonatal Nurs* 2007; **36**(6): 581-91.
4. Hartnett E, Haber J, Catapano P, et al. The Impact of an Interprofessional Pediatric Oral Health Clerkship on Advancing Interprofessional Education Outcomes. *J Dent Educ* 2019; **83**(8): 878-86.
5. Niranjan R, Kim J, Lin B, et al. Pediatric dental education improves interprofessional healthcare students' clinical competence in children's oral health assessment. *Dent J* 2019; **7**(4).
6. Dolce MC, Haber J, Savageau JA, Hartnett E, Riedy CA. Integrating oral health curricula into nurse practitioner graduate programs: Results of a US survey. *J Am Assoc Nurse Pract* 2018; **30**(11): 638-47.
7. Chinn CH. Effectiveness of an oral health program in improving the knowledge and competencies of head start staff. *Pediatr Dent* 2011; **33**(5): 403-8.
8. Braun P, Katina Widmer R, Ling SB, et al. Impact of an interprofessional oral health education program on health care professional and practice behaviors: a RE-AIM analysis. *Pediatric Health Med Ther* 2015; **6**: 101-9.
9. Clark M, Quinonez R, Bowser J, Silk H. Curriculum influence on interdisciplinary oral health education and practice. *J Public Health Dent* 2017; **77**(3): 272-82.
10. McConnell ES, Lekan D, Hebert C, Leatherwood L. Academic-practice partnerships to promote evidence-based practice in long-term care: Oral hygiene care practices as an exemplar. *Nurs Outlook* 2007; **55**(2): 95-105.
11. Bassiouny MA, Tweddale E. Oral health considerations in anorexia and bulimia nervosa: 2. Multidisciplinary management and personalized dental care. *Gen Dent* 2017; **65**(5): 24-31.
12. Bonwell PB, Parsons PL, Best AM, Hise S. An Interprofessional Educational Approach to Oral Health Care in the Geriatric Population. *Gerontol Geriatr Educ* 2014; **35**(2): 182-99.
13. Maxey HL, Norwood CW, Weaver DL. Primary care physician roles in health centers with oral health care units. *J Am Board Fam Med* 2017; **30**(4): 491-504.
14. Murphy KL, Larsson LS. Interprofessional oral health initiative in a nondental, American Indian setting. *J Am Assoc Nurse Pract* 2017; **29**(12): 733-40.
15. Petrosky M, Colaruotolo LA, Billings RJ, Meyerowitz C. The integration of social work into a postgraduate dental training program: A fifteen-year perspective. *J Dent Educ* 2009; **73**(6): 656-64.
16. Nair MK, Renjit M, Siju KE, Leena ML, George B, Kumar GS. Effectiveness of a community oral health awareness program. *Indian Pediatr* 2009; **46 Suppl**: s86-90.
17. Shrivastava R, Couturier Y, Girard F, et al. Appreciative inquiry in evaluating integrated primary oral health services in Quebec Cree communities: a qualitative multiple case study. *BMJ Open* 2020; **10**(6): 1.
18. Haber J. Promoting Oral Health for Mothers and Children: A Nurse Home Visitor Education Program. *Pediatr Nurs* 2020; **46**(2): 70-6.
19. Gold J, Tomar SL. Interdisciplinary Community-Based Oral Health Program for Women and Children at WIC. *Matern Child Health J* 2018; **22**(11): 1617-23.
20. Mouradian WE, Schaad DC, Kim S, et al. Addressing disparities in children's oral health: A dental-medical partnership to train family practice residents. *J Dent Educ* 2003; **67**(8): 886-95.
21. Mowat S, Hein C, Walsh T, MacDonald L, Grymonpre R, Sisler J. Changing Health Professionals' Attitudes and Practice Behaviors Through Interprofessional Continuing Education in Oral-Systemic Health. *J Dent Educ* 2017; **81**(12): 1421-9.
22. Savageau JA, Sullivan KM, Sawosik G, Sullivan E, Silk H. Status of Oral Health Training in U.S. Primary Care Programs: A Qualitative Study to Define Characteristics and Outcomes. *J Dent Educ* 2019; **83**(8): 865-77.
23. Nicolae A, Levin L, Wong PD, et al. Identification of early childhood caries in primary care settings. *Paediatr Child Health (1205-7088)* 2018; **23**(2): 111-5.
24. Scherr S, Idzik S, Williams D. Integrating Oral Health Screening Into Primary Care to Promote Dental Referrals in Maryland. *J Dr Nurs Pract* 2020; **13**(1): 42-63.
25. Saparamadu KD. Prevention of oral diseases in developing countries. *Int Dent J* 1984; **34**(3): 166-9.
26. Wysen KH, Hennessy PM, Lieberman MI, Garland TE, Johnson SM. Kids get care: integrating preventive dental and medical care using a public health case management model. *J Dent Educ* 2004; **68**(5): 522-30.
27. Kimball CEJ. Medical dental collaboration: quality, cost effective health care. *Pa Dent J* 2013; **80**(2): 22-7.

28. Anumanrajadhon T, Rajchagool S, Nitisiri P, et al. The community care model of the Intercountry Centre for Oral Health at Chiangmai, Thailand. *Int Dent J* 1996; **46**(4): 325-33.
29. Markowski AM, Curry Greenwood K, Parker JL, Corkery MB, Dolce MC. A Novel Interprofessional Faculty Approach for Integrating Oral Health Promotion Competencies into a Physical Therapist Curriculum. *J Allied Health* 2018; **47**(1): 19-24.
30. Leone SM, Quinonez RB, Chuang A, et al. Introduction of prenatal oral health into medical students' obstetrics training. *J Dent Educ* 2017; **81**(12): 1405-12.
31. Lavigne SE. Dental hygienists in multidisciplinary health care. *Probe* 1999; **33**(3): 11-4.
32. Clark L. A medical-dental Integration Model. *Dimens Dent Hyg* 2019: 33-5.
33. Janotha BL, Tamari K, Evangelidis-Sakellson V. Dental and Nurse Practitioner Student Attitudes About Collaboration Before and After Interprofessional Clinical Experiences. *J Dent Educ* 2019; **83**(6): 638-44.
34. Kordsmeier JA, Ty Williams C, Anthamatten A. Teamwork and Oral Health in Diabetes Care. *J Dr Nurs Pract* 2020; **13**(1): 17-24.
35. Ivon Falcon C, Coplen AE, Davis-Risen S, Korte D, Fontana M, Furgeson D. Impact of an Interprofessional Education Intervention and Collaborative Practice Agreements of Expanded Practice Dental Hygienists in Oregon. *J Dent Hyg* 2020; **94**(3): 6-15.
36. Nash WA, Hall LA, Lee Ridner S, et al. Evaluation of an interprofessional education program for advanced practice nursing and dental students: The oral-systemic health connection. *Nurse Educ Today* 2018; **66**: 25-32.
37. Berkowitz O, Brisotti MF, Gascon L, Henshaw M, Kaufman LB. The Impact of an Interprofessional Oral Health Curriculum on Trainees. *J Phys Assist Educ* 2017; **28**(1): 2-9.
38. Buck MM, Tilson ER, Andersen JC. Implementation and evaluation of an interdisciplinary health professions core curriculum. *J Allied Health* 1999; **28**(3): 174-8.
39. King J. A multidisciplinary approach... dental health education. *Nurs Times* 1984; **80**(28): 32-3.
40. Lewis CW, Barone L, Quinonez RB, Boulter S, Mouradian WE. Chapter oral health advocates: A nationwide model for pediatrician peer education and advocacy about oral health. *Int J Dent* 2013; **2013**.
41. Olatosi OO, Oladugba A, Oyapero A, et al. A Preexperimental Study to Assess the Impact of an Interdisciplinary Educational Intervention on Nurses' Knowledge of Perinatal and Infant Oral Health Care. *J Int Soc Prev Community Dent* 2019; **9**(6): 619-29.
42. Anderson KL, Smith BS, Maseman DC. Integration of an oral health curriculum into a physician assistant program. *J Allied Health* 2011; **40**(1): 19-24.
43. Thompson BM, Bratzler DW, Fisher MJ, Torres A, Faculty E, Sparks RA. Working together: Using a unique approach to evaluate an interactive and clinic-based longitudinal interprofessional education experience with 13 professions. *J Interprof Care* 2016; **30**(6): 754-61.
44. Mouradian WE, Reeves A, Kim S, et al. A new oral health elective for medical students at the University of Washington. *Teach Learn Med* 2006; **18**(4): 336-42.
45. Lary MJ, Lavigne SE, Muma RD, Jones SE, Hoeft HJ. Breaking down barriers: Multidisciplinary education model. *J Allied Health* 1997; **26**(2): 63-9.
46. Grant L, McKay LK, Rogers LG, Wiesenthal S, Cherney SL, Betts LA. An interprofessional education initiative between students of dental hygiene and bachelor of science in nursing. *Can J Dent Hyg* 2011; **45**(1): 36-44.
47. Dolce MC, Parker JL, Bhalla P, Anderson C. A Cooperative Education Model for Promoting Oral Health and Primary Care Integration within a Health Care for the Homeless Program. *J Health Care Poor Underserved* 2018; **29**(2): 591-600.
48. Karpa K, Graveno M, Brightbill M, et al. Geriatric Assessment in a Primary Care Environment: A Standardized Patient Case Activity for Interprofessional Students. *MedEdPORTAL* 2019; **15**: 10844.
49. Graham E, Negron R, Domoto P, Milgrom P. Children's oral health in the medical curriculum: a collaborative intervention at a university-affiliated hospital. *J Dent Educ* 2003; **67**(3): 338-47.
50. Salama F, Kebriaei A, Rothe V. Effectiveness of a basic training presentation on infant oral health care for family medicineresidents. *Pediatr Dent* 2010; **32**(2): 106-9.
51. Tong N. Collaborative practice: the future for dental hygienists and therapists. *Dent Health* 2015; **54**(3): 29-30.
52. Jordan RA, Sirsch E, Gesch D, Zimmer S, Bartholomeyczik S. Improvement of oral health care in geriatric care by training of nurses and nursing assistants for the elderly. *Pflege* 2012; **25**(2): 97-105.
53. Kobayashi M, Chi D, Coldwell SE, Domoto P, Milgrom P. The effectiveness and estimated costs of the Access to Baby and Child Dentistry program in Washington state. *J Am Dent Assoc* 2005; **136**(9): 1257-63.
54. Macnab AJ, Rozmus J, Benton D, Gagnon FA. 3-year results of a collaborative school-based oral health program in a remote First Nations community. *Rural Remote Health* 2008; **8**(2): 882.

55. Martinez-Mier EA, Soto-Rojas AE, Stelzner SM, Lorant DE, Riner ME, Yoder KM. An international, multidisciplinary, service- learning program: An option in the dental school curriculum. *Education for Health: Change in Learning and Practice* 2011; **24**(1): 1-12.
56. Garland T, Smith L, Fuccillo R. Addressing oral health needs through interprofessional education and practice. *Journal of the California Dental Association* 2014; **42**(10): 701-9.
57. Kendall N. Improving access to oral surgery services in primary care. *Primary Dental Care: Journal of the Faculty of General Dental Practitioners* 2009; **16**(4): 137-42.
58. More FG, Sasson LM, Godfrey EM, Sehl RB. Collaboration between dietetics and dentistry: dietetic internship in pediatric dentistry. *Top Clin Nutr* 2005; **20**(3): 259-68.
59. Mayberry ME, Gonik B, Trombly RM. Perinatal Oral Health: A Novel Collaborative Initiative to Improve Access, Attitudes, Comfort Level, and Knowledge of Pregnant Women and Dental Providers. *AJP Reports* 2020; **10**(1): E54-E61.
60. Rothmund WL, O'Kelley-Wetmore AD, Jones ML, Smith MB. Oral Manifestations of Menopause: An Interprofessional Intervention for Dental Hygiene and Physician Assistant Students. *J Dent Hyg* 2017; **91**(6): 21-32.
61. Tynan A, Deeth L, McKenzie D, et al. Integrated approach to oral health in aged care facilities using oral health practitioners and teledentistry in rural Queensland. *Aust J Rural Health* 2018; **26**(4): 290-4.
62. Rowan MS, Mason M, Robitaille A, Labrecque L, Tocchi CL. An innovative medical and dental hygiene clinic for street youth: Results of a process evaluation. *Eval Program Plann* 2013; **40**: 10-6.
63. Kaufman LB, Henshaw MM, Brown BP, Calabrese JM. Oral Health and Interprofessional Collaborative Practice: Examples of the Team Approach to Geriatric Care. *Dent Clin North Am* 2016; **60**(4): 879-90.
64. McAnulla A, Reid B, Zieba M. Developing an integrated resource to promote oral health in nursing homes. *Nursing Older People (2014+)* 2018; **30**(2): 25.
65. Heuer S, Gance-Cleveland B. Family-centered care. Integrated medical and dental health in primary care. *J Spec Pediatr Nurs* 2007; **12**(1): 61-5.
66. Jackson JT, Quinonez RB, Kerns AK, et al. Implementing a prenatal oral health program through interprofessional collaboration. *J Dent Educ* 2015; **79**(3): 241-8.
67. Mann J, Doshi M. Mouth care matters (MCM) is a health education England training initiative focussed on improving the oral health of older people. The hospital arm of the programme was developed and piloted at east Surrey hospital and is currently being rolled out across all acute trusts in Kent, Surrey and Sussex. *Age and Ageing Conference: British Geriatrics Society Communications to the Spring Meeting* 2017; **46**(Supplement 2).
68. Baker GH, Jr. Integration of Oral and General Health in Maternal and Child Populations. *J Public Health Dent* 1990; **50**(6): 402-5.
69. Allen KL, More FG. Clinical simulation and foundation skills: an integrated multidisciplinary approach to teaching. *J Dent Educ* 2004; **68**(4): 468-74.
70. Manski RJ, Hoffmann D, Rowthorn V. Increasing access to dental and medical care by allowing greater flexibility in scope of practice. *Am J Public Health* 2015; **105**(9): 1755-62.
71. Mattevi GS, de Rossi Figueiredo D, Patrício ZM, da Silva Rath IB. The participation of the dental surgeon in the multidisciplinary health team for child care in the hospital context. *Ciencia e Saude Coletiva* 2011; **16**(10): 4229-36.
72. Dimmick SL, Burgiss SG, Robbins S, Black D, Jarnagin B, Anders M. Outcomes of an integrated telehealth network demonstration project. *Telemed J E Health* 2003; **9**(1): 13-23.
73. Kolisa Y. Assessment of oral health promotion services offered as part of maternal and child health services in the Tshwane Health District, Pretoria, South Africa. *Afr J Prim Health Care Fam Med* 2016; **8**(1).
74. Charteris P, Kinsella T. The Oral Care Link Nurse: A facilitator and educator for maintaining oral health for patients at the Royal Hospital for Neuro-disability. *Spec Care Dentist* 2001; **21**(2): 68-71.
75. Atchison KA, Weintraub JA. Integrating Oral Health and Primary Care in the Changing Health Care Landscape. *N C Med J* 2017; **78**(6): 406-9.
76. Jeganathan S, Purnomo J, Houtzager L, Batterham M, Begley K. Development and validation of a three-item questionnaire for dietitians to screen for poor oral health in people living with human immunodeficiency virus and facilitate dental referral. *Nutr Diet* 2010; **67**(3): 177-81.
77. Connor A, Rainer LP, Simcox JB, Thomisee K. Increasing the delivery of health care services to migrant farm worker families through a community partnership model. *Public Health Nurs* 2007; **24**(4): 355-60.
78. Davis MM, Hilton TJ, Benson S, et al. Unmet dental needs in rural primary care: a clinic-, community-, and practice-based research network collaborative. *J Am Board Fam Med* 2010; **23**(4): 514-22.
79. Atchison KA, Rozier RG, Weintraub JA. Integration of Oral Health and Primary Care: Communication, Coordination and Referral. Discussion Paper, National Academy of Medicine, Washington, DC. *NAM Perspectives* 2018.

80. Atchison KA, Weintraub JA, Rozier RG. Bridging the dental-medical divide: Case studies integrating oral health care and primary health care. *J Am Dent Assoc* 2018; **149**(10): 850-8.
81. Rabiei M, Yousefbeyk F, Ghasemi S, Pirzamanbin S. An investigation on How Community Pharmacies and Herbalist's Shops manage patients with probable Oral Cancer. *J Res Dent Maxillofac Sci* 2016; **1**(2): 1-6
82. Previato GF, Baldissera VDA. Portraits of interprofessional collaborative practice in the primary health care teams. *Rev Gaucha Enferm* 2018; **39**: e20170132-e.
83. Reeves S, Pryce A. Emerging themes: An exploratory research project of an interprofessional education module for medical, dental and nursing students. *Nurse Educ Today* 1998; **18**(7): 534-41.
84. Mann PC. Training future dental clinicians. An integrated approach to health care. *N Y State Dent J* 2009; **75**(5): 32-5.
85. Myers-Wright N, Lamster IB. A New Practice Approach for Oral Health Professionals. *J Evid Based Dent Pract* 2016; **16**: 43-51.
86. Merrick J, Shapira J. Preventive dental health for persons with Down syndrome. *Int J Adolesc Med Health* 2000; **12**(1): 81-4.
87. Makuch A, Reschke K. Playing games in promoting childhood dental health. *Patient Educ Couns* 2001; **43**(1): 105-10.
88. Bowen WH. Trends in prevention--promotion of oral health within general health care... Possibilities and limitations in preventive dentistry. Proceedings of the 4th World Congress on Preventive Dentistry. Umeå, Sweden, September 3-5, 1993. *Adv Dent Res* 1995; **9**(2): 77-151.
89. Alem I, Goodson S, Armijo D, Pratap S. Breaking down oral health care siloes with innovative technology. *Pediatrics* 2017; **142**(1).
90. Clarke SJ. The impact of a community health advisor-based intervention on self-reported frequency of dental visits in a rural, low-income African American Alabama community: The University of Alabama at Birmingham; 2007.
91. Edwards J. Improving oral care for at-risk children: integrating oral health into the CDC model. *Access* 2010; **24**(2): 16-9.
92. Lapidos A, Gwozdek A. An Interprofessional Approach to Exploring the Social Determinants of Health with Dental Hygiene Students. *J Allied Health* 2016; **45**(3): e43-7.
93. Sullivan A, Giroux P. Enhanced Care Through Interprofessional Education Among Dental Hygiene and Occupational Therapy Students. *J Harmoniz Res Appl Sci* 2016; **4**(4): 160-163.
94. Taylor E, Marino D, Biordi D, et al. Improving the Oral Health Status of Children: A Pilot Intervention at an Urban and Rural WIC Program in Northeastern Ohio. *J Am Diet Assoc* 2011; **111**(9).
95. Vandamme K, Opdebeeck H, Naert I. Pathways in multidisciplinary oral health care as a tool to improve clinical performance. *Int J Prosthodont* 2006; **19**(3): 227-35.
96. Olatosi OO, Oladugba A, Oyapero A, et al. Erratum: A preexperimental study to assess the impact of an interdisciplinary educational intervention on nurses' knowledge of perinatal and infant oral health care *J Int Soc Prev Community Dent* 2020; **10**(2): 236.
97. Collins E, Fair N, Dickinson A, Peacock K. Collaboration between primary and secondary/tertiary services in oral health. *Prim Health Care* 2009; **19**(1): 35-9.
98. Dounis G, Ditmyer M, VanBeuge S, et al. Interprofessional faculty development: Integration of oral health into the geriatric diabetes curriculum, from theory to practice. *J Multidiscip Healthc* 2013; **7**.
99. Duley SI, Fitzpatrick P. An oral health survey instrument to assist the bariatric nurse in the early determination of suitable surgery candidacy. *Bariatr Nurs Surg Patient Care* 2009; **4**(4): 307-16.
100. Dsouza R, Quinonez R, Hubbell S, Brame J. Promoting oral health in nursing education through interprofessional collaborative practice: A quasi-experimental survey study design. *Nurse Educ Today* 2019; **82**: 93-8.
101. Czarnecki GA, Kloostra SJ, Boynton JR, Inglehart MR. Nursing and dental students' and pediatric dentistry residents' responses to experiences with interprofessional education. *J Dent Educ* 2014; **78**(9): 1301-12.
102. Cooper D, Kim J, Duderstadt K, Stewart R, Lin B, Alkon A. Interprofessional oral health education improves knowledge, confidence, and practice for pediatric healthcare providers. *Frontiers in Public Health* 2017; **5**(AUG).
103. Byrd MG, Quinonez RB, Lipp K, Chuang A, Phillips C, Weintraub JA. Translating prenatal oral health clinical standards into dental education: results and policy implications. *J Public Health Dent* 2019; **79**(1): 25-33.
104. Boynes SG, Lauer A, Deutchman M, Martin AB. An Assessment of Participant-Described Interprofessional Oral Health Referral Systems Across Rurality. *J Rural Health* 2017; **33**(4): 427-37.
105. Bowser J, Sivahop J, Glicken A. Advancing Oral Health in Physician Assistant Education: Evaluation of an Innovative Interprofessional Oral Health Curriculum. *J Physician Assist Educ* 2013; **24**(3): 27-30.

106. Berg JH, Stapleton FB. Physician and dentist: New initiatives to jointly mitigate early childhood oral disease. *Clin Pediatr* 2012; **51**(6): 531-7.
107. Anders PL, Scherer YK, Hatton M, Antonson D, Austin-Ketch T, Campbell-Heider N. Using standardized patients to teach interprofessional competencies to dental students. *J Dent Educ* 2016; **80**(1): 65-72.
108. Allen HBRDHM, Gunaldo TPPDPTMHS, Schwartz ERDHB. Creating Awareness for the Social Determinants of Health: Dental hygiene and nursing student interprofessional service-learning experiences. *J Dent Hyg* 2019; **93**(3): 22-8.
109. Nelson JD, Spencer SM. By word of mouth: A qualitative approach to understanding the integration of preventive dental health in primary care settings. 2015; (3704377): 141.
110. Meeks VI, Johnson MN, Salzman RE, Yoon S. Developing an oral health care curriculum for Rwandan primary school-aged children using the classroom teachers in a non-traditional manner to promote life-long oral health knowledge and practices. *Ann Glob Health* 2016; **82 (3)**: 482.
111. A OR, Langan C. Whose cares about mouth care? An inter-disciplinary quality improvement initiative in a large acute teaching hospital. *Dysphagia* 2019; **34 (5)**: 777-8.
112. Khokhar WA, Williams KC, Odeyemi O, Clarke T, Tarrant CJ, Clifton A. Open wide: a dental health and toothbrush exchange project at an inpatient recovery and rehabilitation unit. *Ment Health Rev* 2011; **16**(1): 36-41.
113. Forbes J, Sierra T, Papa J. Advancing oral health knowledge and attitudes of physician assistant students using the smiles for life oral health curriculum. *Fam Med* 2018; **50**(10): 775-8.
114. Golinveaux J, Gerbert B, Cheng J, et al. Oral health education for pediatric nurse practitioner students. *J Dent Educ* 2013; **77**(5): 581-90.
115. Haber J, Spielman AI, Wolff M, Shelley D. Interprofessional education between dentistry and nursing: the NYU experience. *J Calif Dent Assoc* 2014; **42**(1): 44-51.
116. Haber J, Hartnett E, Allen K, et al. Putting the Mouth Back in the Head: HEENT to HEENOT. *Am J Public Health* 2015; **105**(3): 437-41.
117. Hahn JE, Fitzgerald L, Markham YK, Glassman P, Guenther N. Infusing oral health care into nursing curriculum: addressing preventive health in aging and disability. *Nurs Res Pract* 2012; **2012**: 157874.
118. Haresaku S, Miyoshi M, Kubota K, et al. Effect of interprofessional education on oral assessment performance of nursing students. *Clin Exp Dent Res* 2020; **6**(1): 51-8.
119. Heath J, Aker R, Feld H, Singer RL, Norton J. A pilot interprofessional program to promote oral health and wellness in Appalachian children. *J Prof Nurs* 2019; **35**(5): 412-6.
120. Braun PA, Cusick A. Collaboration Between Medical Providers and Dental Hygienists in Pediatric Health Care. *J Evid Based Dent Pract* 2016; **16**: 59-67.
121. Chung R, Shu-Yin JL, Abel SN, et al. HIV screening in the dental setting in New York State. *PLoS One* 2020; **15**(4).
122. Biethman RK, Pandarakalam C, Garcia MN, Whitener S, Hildebolt CF. Screening for Diabetes in a Dental School Clinic to Assess Interprofessional Communication Between Physicians and Dental Students. *J Dent Educ* 2017; **81**(9): 1062-7.
123. Beaglehole RH, Beaglehole R. Promoting radical action for global oral health: integration or independence? *The Lancet* 2019; **394**(10194): 196-8.
124. Larson AK. The early childhood caries prevention program in Palau. *Pac Health Dialog* 2003; **10**(1): 106-10.
125. Stormon N, Pradhan A, McAuliffe A, Ford PJ. Does a facilitated pathway improve access to dental services for homeless and disadvantaged adults? *Eval Program Plann* 2018; **71**: 46-50.
126. Simon LE, Eve EJ, Dolce MC, Allareddy V, Nalliah RP. Physician Assistant Student Perceptions of an Interprofessional, Peer-to-Peer Oral Health Curriculum Led by Dental Students. *J Physician Assist Educ (Lippincott Williams & Wilkins)* 2017; **28**(4): 210-3.
127. NewsRx. Dentistry; Study Findings from New York University Provide New Insights into Dentistry (Interdisciplinary Community-Based Oral Health Program for Women and Children at WIC). *Health Insurance Law Weekly* 2019: 14.
128. Shiel H, Grogan A, Dougall A. The implementation and evaluation of an oral health risk assessment by non-dental personnel for patients with bleeding disorders. *Haemophilia* 2012; **18**: 133.
129. Mallonee LF. The need for inter-professional collaboration. *J Dent Hyg* 2012; **86**(2): 56-7.
130. Berge JSRDHREFM. The Dental and Mental Health Connection: Integrating New Dental Workforce Strategies in Minnesota. *J Dent Hyg (Online)* 2013; **87**(6): 398-9.
131. Giddon DB. Medical-dental integration. *J Am Dent Assoc* 2018; **149**(9): 750-.
132. Marshall SE, Kunzel C, Lamster IB, Bin C, Northridge ME, Huang C. Integrating Oral and General Health Screening at Senior Centers for Minority Elders. *Am J Public Health* 2013; **103**(6): 1022-5.

133. Norwood CW, Maxey HL, Randolph C, Gano L, Kochhar K. Administrative Challenges to the Integration of Oral Health With Primary Care: A SWOT Analysis of Health Care Executives at Federally Qualified Health Centers. *J Ambul Care Manage* 2017; **40**(3): 204-13.
134. Barsh LI. The Social Web and medical/dental education. *Sleep Breath* 2011; **15**(2): 237-8.
135. Nishina M, Hoshino T, Murai K, Miyata K, Ishii Y, Saito A. Effect of collaborative intervention by medical and dental professionals on adherence to smoking abstinence. *Bull Tokyo Dent Coll* 2015; **56**(1): 57-61.
136. Donoff B, McDonough JE, Riedy CA. Integrating oral and general health care. *N Engl J Med* 2014; **371**(24): 2247-9.
137. Bansal K, Kharbanda O, Sharma J, et al. Effectiveness of an integrated perinatal oral health assessment and promotion program on the knowledge in Indian pregnant women. *J Indian Soc Pedod Prev Dent* 2019; **37**(4): 383-91.
138. Pucca GA, Gabriel M, de Araujo ME, de Almeida FCS. Ten Years of a National Oral Health Policy in Brazil: Innovation, Boldness, and Numerous Challenges. *J Dent Res* 2015; **94**(10): 1333-7.
139. Myers Virtue S, Rotz ME, Boyd M, Lykon JL, Waldron EM, Theodorou J. Impact of a novel interprofessional dental and pharmacy student tobacco cessation education programme on dental patient outcomes. *J Interprof Care* 2018; **32**(1): 52-62.
140. Janssen JA, Lampiris LN. Disaster Response in Illinois: The Role for Dentists and Dental Hygienists. *Dent Clin North Am* 2007; **51**(4): 779-84.
141. McClure JB, Blasi PR, Cook A, et al. Oral health 4 life: Design and methods of a semi-pragmatic randomized trial to promote oral health care and smoking abstinence among tobacco quitline callers. *Contemp Clin Trials* 2017; **57**: 90-7.
142. Kateeb ET, McKernan SC, Gaeth GJ, Kuthy RA, Adrianse NB, Damiano PC. Predicting dentists' decisions: a choice-based conjoint analysis of Medicaid participation. *J Public Health Dent* 2016; **76**(3): 171-8.
143. Jones ML, Boyd LD. Interface with a Community Feeding Team to Address Oral Health of Special Needs Children: A Pilot Project. *J Dent Hyg* 2011; **85**(2): 132-42.
144. Laura LM, Nicole A, Elizabeth S, Sheryl S, Heather JD. Development of an Interprofessional Diabetes and Oral Hygiene Education Program for Youth with Type 2 Diabetes Mellitus. *Can J Diabetes* 2012; **36**(6): 327-31.
145. Goyal A, Grover A, Gauba K, et al. A community-based pragmatic, controlled trial for preventing and reducing oral diseases among 1-6-year-old children visiting Anganwadi centers, under the Integrated Child Development Scheme, India. *BMC Public Health* 2019; **19**(1).
146. Taylor E, Marino D, Thacker S, DiMarco M, Huff M, Biordi D. Expanding oral health preventative services for young children: A successful interprofessional model. *J Allied Health* 2014; **43**(1): e5-e9.
147. Obana M, Furuya J, Matsubara C, et al. Effect of a collaborative transdisciplinary team approach on oral health status in acute stroke patients. *J Oral Rehabil* 2019; **46**(12): 1170-6.
148. Ho J, Lai YH, Benton D, Duffy D, Harrison R, Macnab AJ. Evaluation of an oral health promotion program in a remote first nations community. *J Investig Med* 2005; **53**(1).
149. Rosedale MT, Strauss SM. Diabetes screening at the periodontal visit: Patient and provider experiences with two screening approaches. *Int J Dent Hyg* 2012; **10**(4): 250-8.
150. Antonarakis GS. Integrating dental health into a family-oriented health promotion approach in guatemala. *Health Promot Pract* 2011; **12**(1): 79-85.
151. Lau P, Meethal C, Middleton M, Clark M, Darby I. 'Say Ahhh': What do dentists, general medical practitioners and community pharmacists do about halitosis? *Int Dent J* 2019; **69**(4): 311-20.
152. Vallabhan MK, Jimenez EY, McCauley G, Kong AS. 140. Early Lessons Learned from Implementing Telehealth in Rural School Based Health Centers. *J Adolesc Health* 2020; **66** (suppl 2): S71-S2.
153. Crall JJ, Pourat N, Inkelas M, Lampron C, Scoville R. Improving The Oral Health Care Capacity Of Federally Qualified Health Centers. *Health Aff* 2016; **35**(12): 2216-23.
154. Thomson A, Dickenson AJ, Ross-Russell M. Integrated care: a new model for dental education. *Br Dent J* 2021; **231**(3): 187-90.
155. Niesten D, Gerritsen AE, Leve V. Barriers and facilitators to integrate oral health care for older adults in general (basic) care in East Netherlands. Part 1: Normative integration. *Gerodontology* 2021; **38**(2): 154-65.
156. Niesten D, Gerritsen AE, Leve V. Barriers and Facilitators to integrate Oral Health care for Older Adults in General (Basic) Care in East Netherlands. Part 2 Functional Integration. *Gerodontology* 2021; **38**(3): 289-99.
157. Pourat N, Martinez AE, Haley LA, Crall JJ. Colocation Does Not Equal Integration: Identifying and Measuring Best Practices in Primary Care Integration of Children's Oral Health Services in Health Centers. *J Evid Based Dent Pract* 2020; **20**(4): 101469.
158. Gesko DS, Worley D, Rindal BD. Creating systems aligned with the triple-aim and value-based care. *J Public Health Dent* 2020; **80** Suppl 2: S109-S13.

159. Dolce MC, Barrow J, Jivraj A, Pham D, Da Silva JD. Interprofessional value-based health care: Nurse practitioner-dentist model. *J Public Health Dent* 2020; **80** Suppl 2: S44-S9.
160. Greiner AC, Duhl Glicken A. What Primary Care Innovation Teaches Us About Oral Health Integration? *AMA J Ethics* 2022; **24**(1): E64-72.
161. Tabrizi M, Lee WC. A Pilot Study of an Interprofessional Program Involving Dental, Medical, Nursing, and Pharmacy Students. *Front Public Health* 2020; **8**: 602957.
162. Pike NA, Kinsler JJ, Peterson JK, et al. Improved oral health knowledge in a primary care pediatric nurse practitioner program. *J Am Assoc Nurse Pract* 2022; **34**(5): 755-62.
163. Adeniyi A, Donnelly L, Janssen P, Jevitt C, Von Bergmann H, Brondani M. An interprofessional model of care for oral health during pregnancy. *J Interprof Care* 2022: 1-9.
164. Ramos, D.V.R., Miraglia, J.L., Monteiro, C.N. et al. Risk assessment for oral urgent treatment in Primary Healthcare: a cross-sectional study. BMC Health Serv Res 20, 1012 (2020).
165. Linabarger M, Brown M, Patel N. A Pilot Study of Integration of Medical and Dental Care in 6 States. Prev Chronic Dis 2021;18:210027.
166. Mason DK, Gibson J, Devennie JC, Haughney MG, Macpherson LM. Integration of primary care dental and medical services: A pilot investigation. Br Dent J 1994; 177(8): 283-6.
167. O'Neil M, Clarkson H. 'Reaching families with young children': a community dental health project for preventing early childhood caries. Probe (08341494) 2002; 36(4): 145-8.
168. Lawrence HP, Romanetz M, Rutherford L, Cappel L, Binguis D, Rogers JB. Effects of a community-based prenatal nutrition program on the oral health of Aboriginal preschool children in northern Ontario. Probe (08341494) 2004; 38(4): 172-88.
169. Fallon T, Buikstra E, Cameron M, et al. Implementation of oral health recommendations into two residential aged care facilities in a regional Australian city. Int J Evid Based Healthc 2006; 4(3): 162-79.
170. Niiranen T, Widström E, Niskanen T. Oral Health Care Reform in Finland - Aiming to reduce inequity in care provision. BMC Oral Health 2008; 8(1).
171. Mofidi M, Gambrell A. Community-based dental partnerships: Improving access to dental care for persons living with HIV/AIDS. J Dent Educ 2009; 73(11): 1247-59.
172. Silk H, Gusha J, Adler B, et al. The Central Massachusetts Oral Health Initiative (CMOHI): A successful public-private community health collaboration. J Public Health Dent 2010; 70(4): 308-12.
173. Skapetis T, Gerzina T, Hu W. Can a four-hour interactive workshop on the management of dental emergencies be effective in improving self reported levels of clinician proficiency? Australas Emerg Nurs J 2012; 15(1): 14-22.
174. Vichayanrat T, Steckler A, Tanasugarn C, Lexomboon D. The evaluation of a multi-level oral health intervention to improve oral health practices among caregivers of preschool children. Southeast Asian J Trop Med Public Health 2012; 43(2): 526-39.
175. McKeown LL, Woodbeck HH, Lloyd M. A journey to improve oral care with best practices in long-term care. Can J Dent Hyg 2014; 48(2): 57-62.
176. Biordi DL, Heitzer M, Mundy E, et al. Improving access and provision of preventive oral health care for very young, poor, and low-income children through a new interdisciplinary partnership. Am J Public Health 2015; 105: e26-e9.
177. De Visschere L, de Baat C, De Meyer L, et al. The integration of oral health care into day-to-day care in nursing homes: a qualitative study. Gerodontology 2015; 32(2): 115-22.
178. Heilbrunn-Lang AY, de Silva AM, Lang G, et al. Midwives' perspectives of their ability to promote the oral health of pregnant women in Victoria, Australia. BMC Pregnancy Childbirth 2015; 15(1): 110.
179. Kranz AM, Preisser JS, Rozier RG. Effects of Physician-Based Preventive Oral Health Services on Dental Caries. Pediatrics 2015; 136(1): 107-14.
180. McNally M, Martin-Misener R, McNeil K, et al. Implementing oral care practices and policy into long-term care: The brushing up on mouth care project. J Am Med Dir Assoc 2015; 16(3): 200-7.
181. Crall JJ, Illum J, Martinez A, Pourat N. An Innovative Project Breaks Down Barriers to Oral Health Care for Vulnerable Young Children in Los Angeles County. Policy Brief UCLA Cent Health Policy Res 2016; (PB20165): 1-8.
182. de Mey L, Çömlekçi C, de Reuver F, et al. Oral Hygiene in Patients With Severe Mental Illness: A Pilot Study on the Collaboration Between Oral Hygienists and Mental Health Nurses. Perspect Psychiatr Care 2016; 52(3): 194-200.
183. Dooley D, Moultrie NM, Heckman B, Gansky SA, Potter MB, Walsh MM. Oral health prevention and toddler well-child care: Routine integration in a safety net system. Pediatrics 2016; 137(1).
184. George A, Lang G, Johnson M, et al. The evaluation of an oral health education program for midwives in Australia. Women Birth 2016; 29(3): 208-13.
185. Vece L, Sutter R, Sutter C, Toulouse C. Impacting Vulnerable Populations Through Integrating Oral Health Care Into Nurse-managed Health Centers. J Nurse Pract 2016; 12(9): 629-34.

186. Adams SH, Gregorich SE, Rising SS, Hutchison M, Chung LH. Integrating a Nurse-Midwife-Led Oral Health Intervention Into CenteringPregnancy Prenatal Care: Results of a Pilot Study. J Midwifery Womens Health 2017; 62(4): 463-9.
187. Duijster D, Monse B, Dimaisip-Nabuab J, et al. 'Fit for school' – a school-based water, sanitation and hygiene programme to improve child health: Results from a longitudinal study in Cambodia, Indonesia and Lao PDR. BMC Public Health 2017; 17(1): 302.
188. Kohli R, Nelson S, Ulrich S, Finch T, Hall K, Schwarz E. Dental care practices and oral health training for professional caregivers in long-term care facilities: An interdisciplinary approach to address oral health disparities. Geriatric Nursing 2017; 38(4): 296-301.
189. Mathu-Muju KR, McLeod J, Donnelly L, Harrison R, MacEntee MI. The perceptions of first nation participants in a community oral health initiative. Int J Circumpolar Health 2017; 76(1).
190. Sengupta N, Nanavati S, Cericola M, Simon L. Oral Health Integration Into a Pediatric Practice and Coordination of Referrals to a Colocated Dental Home at a Federally Qualified Health Center. Am J Public Health 2017; 107(10): 1627-9.
191. Wright FAC, Law G, Chu SKY, Cullen JS, Le Couteur DG, Chu SKY. Residential age care and domiciliary oral health services: Reach-OHT-The development of a metropolitan oral health programme in Sydney, Australia. Gerodontology 2017; 34(4): 420-6.
192. Batra M, Shah A, Virtanen J, Shah AF, Virtanen JI. Integration of oral health in primary health care through motivational interviewing for mothers of young children: A pilot study. J Indian Soc Pedod Prev Dent 2018; 36(1): 86-92.
193. Burgette JM, Preisser JS, Rozier G. Access to preventive services after the integration of oral health care into early childhood education and medical care. J Am Dent Assoc 2018; 149(12): 1023-31.
194. George A, Dahlen HG, Blinkhorn A, et al. Evaluation of a midwifery initiated oral health-dental service program to improve oral health and birth outcomes for pregnant women: A multi-centre randomised controlled trial. Int J Nurs Stud 2018; 82: 49-57.
195. Janssens B, Vanobbergen J, Lambert M, Schols JMGA, De Visschere L. Effect of an oral healthcare programme on care staff knowledge and attitude regarding oral health: a non-randomised intervention trial. Clin Oral Investig 2018; 22(1): 281-92.
196. Lambert M. Dental attendance in undocumented immigrants before and after the implementation of a personal assistance program: A cross-sectional observational study. Dent J (Basel) 2018; 6(4).
197. Nelson JD, Spencer SM, Blake CE, Moore JB, Martin AB. Elevating Oral Health Interprofessional Practice Among Pediatricians Through a Statewide Quality Improvement Learning Collaborative. J Public Health Manag Pract 2018; 24(3): e19-e24.
198. Simon L, Ji YD, Bell R, et al. Integration of an Oral Health Team into a Student-Faculty Collaborative Clinic: Successes and Challenges. J Health Care Poor Underserved 2018; 29(2): 573-80.
199. Trudnak Fowler T, Matthews G, Black C, Crosby Kowal H, Vodicka P, Edgerton E. Evaluation of a Comprehensive Oral Health Services Program in School-Based Health Centers. Matern Child Health J 2018; 22(7): 998-1007.
200. Tynan A, Deeth L, McKenzie D. An integrated oral health program for rural residential aged care facilities: A mixed methods comparative study. BMC Health Serv Res 2018; 18(1).
201. Ajwani S, Sousa MS, Villarosa AC, et al. Process evaluation of the midwifery initiated oral health-dental service program: Perceptions of dental professionals. Health Promot J Austr 2019; 30(3): 333-43.
202. Basso MB, Nunes NB, Corrêa LBC, Vieira CN, da Silva Vilarinho JLP, Júnior GAP. The construction of the oral health care network in the Federal District, Brazil. Cien Saude Colet 2019; 24(6): 2155-65.
203. Dahlen HG, Johnson M, Hoolsema J, et al. Process evaluation of the midwifery initiated oral health-dental service program: Perceptions of midwives in Greater Western Sydney, Australia. Women Birth 2019; 32(2): e159-e65.
204. George A, Villarosa A, Patterson Norrie T, et al. Process evaluation of the midwifery initiated oral health-dental service program: perceptions of pregnant women. Aust Dent J 2019; 64(1): 55-65.
205. Villena RS, Pesaressi E, Frencken JE. Reducing carious lesions during the first 4 years of life: An interprofessional approach. J Am Dent Assoc 2019; 150(12): 1004-14.
206. Aagaard K, Meléndez-Torres GJ, Overgaard C. Improving oral health in nursing home residents: A process evaluation of a shared oral care intervention. J Clin Nurs 2020.
207. Aronoff-Spencer E, Asgari P, Finlayson TL, et al. A comprehensive assessment for community-based, person-centered care for older adults. BMC Geriatr 2020; 20(1): 193
208. Kanan C, Ohrenberger K, Bayham M, Raskin SE, Tranby EP, Boynes S. MORE Care: an evaluation of an interprofessional oral health quality improvement initiative. J Public Health Dent 2020; 80 Suppl 2: S58-S70.
209. Song PH, White BA, Chisolm DJ, Berney S, Domino ME. The effect of an accountable care organization on dental care for children with disabilities. J Public Health Dent 2020; 80(3): 244– 9.

210. Wood M, Gurenlian J, Freudenthal J, Cartwright E. Interprofessional Health Care Delivery: Perceptions of oral health care integration in a Federally Qualified Health Center. J Dent Hyg 2020; 94(6): 49-55.
211. Arif UA, Pitts E, Farrell C, Fontana M, Kinney JA. Perception and Utilization of Oral Screenings and Fluoride Application in Medical Offices Following the Michigan Caries Prevention Program Training. J Dent Hyg 2021; 95(1): 50-6.
212. Brannemo I, Dahllof G, Cunha Soares F, Tsilingaridis G. Impact of an extended postnatal home visiting programme on oral health among children in a disadvantaged area of Stockholm, Sweden. Acta Paediatr 2021; 110(1): 230-6
213. Ho BV, van der Maarel-Wierink CD, Rollman A, Weijenberg RAF, Lobbezoo F. 'Don't forget the mouth!': a process evaluation of a public oral health project in community-dwelling frail older people. BMC Oral Health 2021; 21(1): 536.
214. Pawloski C, Hilgert J, Senturia K, Davis S, Koday M, Cunha-Cruz J. Medical-Dental Integration in a Rural Community Health Center: A Qualitative Program Evaluation. Health Promot Pract 2022; 23(3): 416-24.
